# In Vitro Demonstration of Human Lipoyl Synthase Catalytic Activity in the Presence of NFU1

**DOI:** 10.1021/acsbiomedchemau.2c00020

**Published:** 2022-06-13

**Authors:** Douglas M. Warui, Debangsu Sil, Kyung-Hoon Lee, Syam Sundar Neti, Olga A. Esakova, Hayley L. Knox, Carsten Krebs, Squire J. Booker

**Affiliations:** ^†^Department of Chemistry and ^‡^Biochemistry and Molecular Biology and the ^§^Howard Hughes Medical Institute, The Pennsylvania State University, University Park, Pennsylvania 16802, United States

**Keywords:** lipoic acid, *S*-adenosylmethionine, iron−sulfur clusters, NFU1, multiple mitochondrial dysfunctions syndrome, radical SAM, lipoyl synthase, LIAS

## Abstract

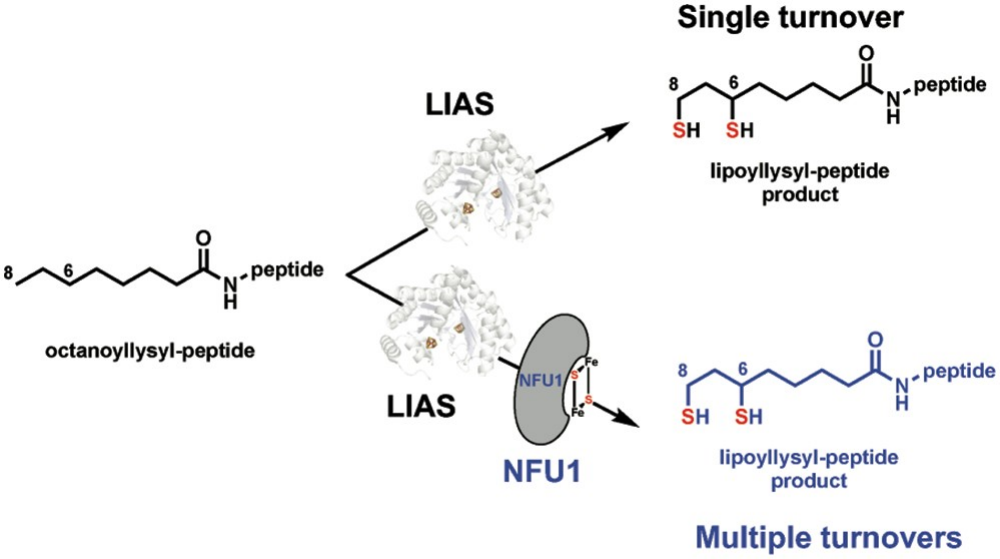

Lipoyl synthase (LS) catalyzes the last step in the biosynthesis of the lipoyl cofactor, which is the attachment of sulfur atoms at C6 and C8 of an *n*-octanoyllysyl side chain of a lipoyl carrier protein (LCP). The protein is a member of the radical *S*-adenosylmethionine (SAM) superfamily of enzymes, which use SAM as a precursor to a 5′-deoxyadenosyl 5′-radical (5′-dA·). The role of the 5′-dA· in the LS reaction is to abstract hydrogen atoms from C6 and C8 of the octanoyl moiety of the substrate to initiate subsequent sulfur attachment. All radical SAM enzymes have at least one [4Fe–4S] cluster that is used in the reductive cleavage of SAM to generate the 5′-dA·; however, LSs contain an additional auxiliary [4Fe–4S] cluster from which sulfur atoms are extracted during turnover, leading to degradation of the cluster. Therefore, these enzymes catalyze only 1 turnover in the absence of a system that restores the auxiliary cluster. In *Escherichia coli*, the auxiliary cluster of LS can be regenerated by the iron–sulfur (Fe–S) cluster carrier protein NfuA as fast as catalysis takes place, and less efficiently by IscU. NFU1 is the human ortholog of *E. coli* NfuA and has been shown to interact directly with human LS (i.e., LIAS) in yeast two-hybrid analyses. Herein, we show that NFU1 and LIAS form a tight complex in vitro and that NFU1 can efficiently restore the auxiliary cluster of LIAS during turnover. We also show that BOLA3, previously identified as being critical in the biosynthesis of the lipoyl cofactor in humans and *Saccharomyces cerevisiae*, has no direct effect on Fe–S cluster transfer from NFU1 or GLRX5 to LIAS. Further, we show that ISCA1 and ISCA2 can enhance LIAS turnover, but only slightly.

## Introduction

**Figure 1:**
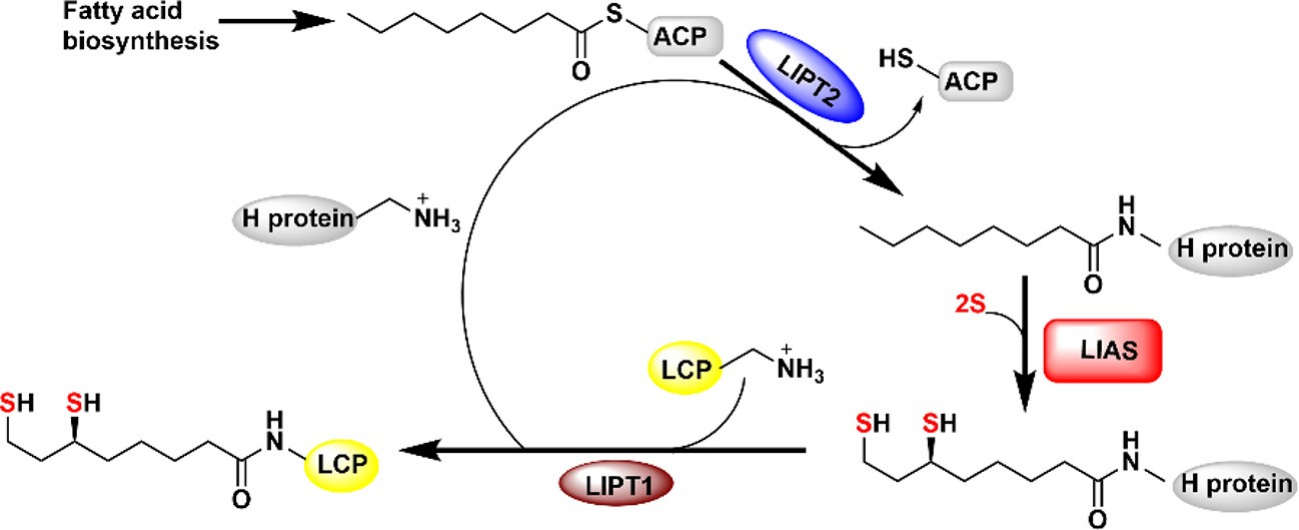
Proposed de novo biosynthetic scheme of lipoyl cofactor in humans.

Lipoic acid is an eight-carbon straight-chain fatty acid containing sulfur atoms at C6 and C8.^1−3^ It is found in all domains of life.^4−11^ Its primary cellular function is as a cofactor in several multienzyme complexes that are involved in energy metabolism and the catabolism of certain amino acids.^7,11^ In humans, these complexes include the pyruvate dehydrogenase complex (PDC), the α-ketoglutarate dehydrogenase complex (KGC), the branched chain oxo-acid dehydrogenase complex (BCODC), the glycine cleavage system (GCS), and the α-ketoadipate complex (KAC), all of which are found in the mitochondrion.^12,13^ In each of these complexes, lipoic acid is tethered covalently in an amide linkage to a target lysyl residue of a lipoyl carrier protein (LCP), producing a 14 Å flexible appendage that can access active sites of other component proteins. Very little free lipoic acid exists in the cell in the absence of supplementation. In fact, the molecule is biosynthesized in its cofactor form rather than as the free acid. This biosynthetic pathway involves a bacterial-type acyl carrier protein (ACP) upon which the C8 fatty acyl backbone is constructed. In humans, LIPT2, an octanoyltransferase, is believed to transfer the octanoyl chain from octanoyl–ACP to the target lysyl residue only of the H protein, the LCP of the GCS. Next, lipoyl synthase (LS, LIAS in humans and LipA in *Escherichia coli*) attaches thiol groups at C6 first, and then at C8, to give the intact lipoyl cofactor.^13,14^ Finally, LIPT1, a lipoyltransferase, is believed to distribute the lipoyl appendage to other LCPs^13,15^ (Figure 1). This biosynthetic pathway differs from the canonical pathway in *E. coli* wherein octanoyltransferase transfers an octanoyl group directly to each of the LCPs, which include the H protein of the GCS and the E2 subunits of the KGC and PDC.^16−21^ LipA then acts on each of the octanoyllysyl–LCPs to generate the respective lipoyl cofactor.^22−24^

**Figure 2:**
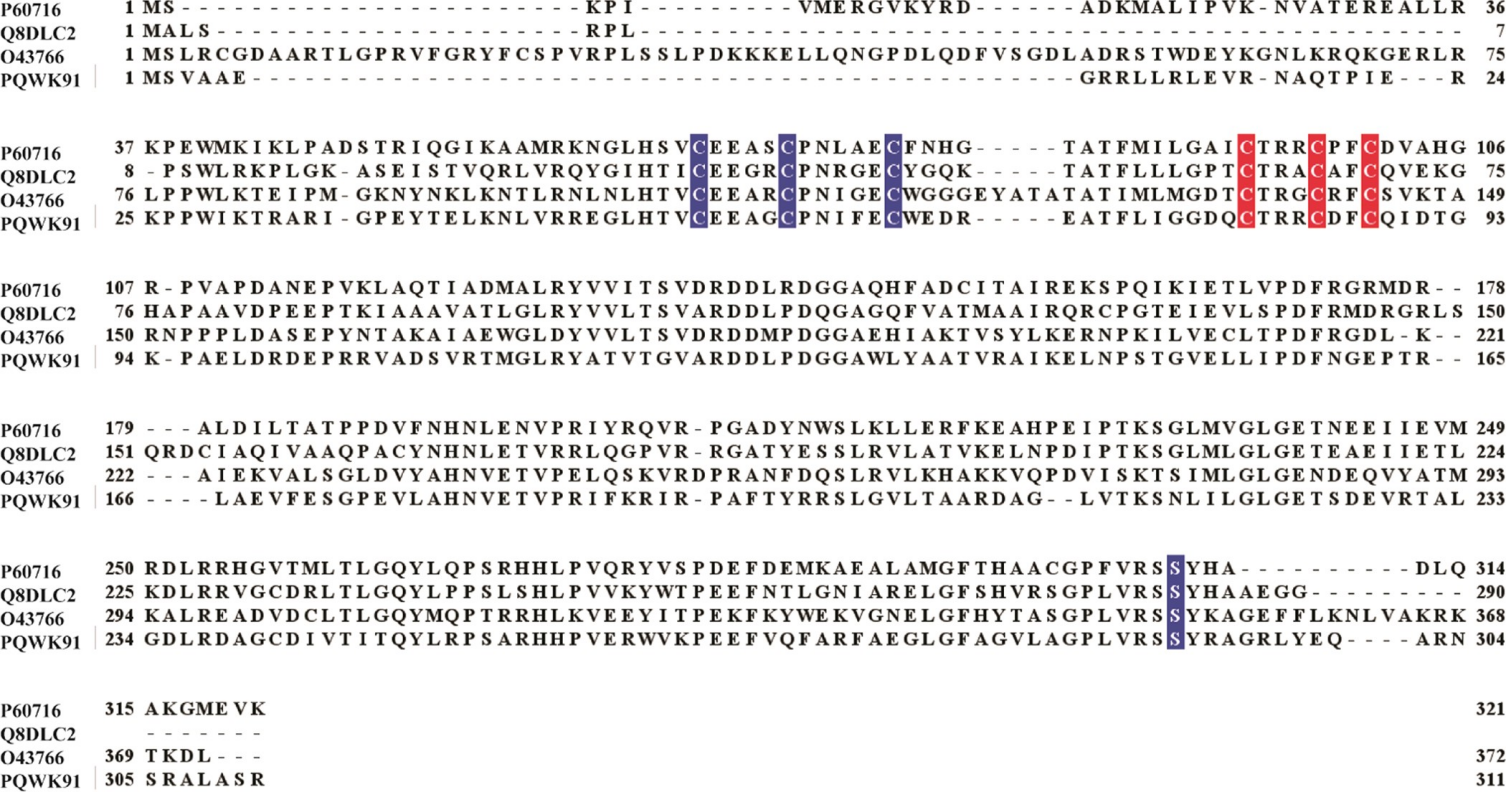
Sequence alignment of LS proteins from *E. coli* (P60716), *M. tuberculosis* (P9WK91), *T. elongatus* (Q8DLC2), and humans (O43766). The conserved residues that ligate the auxiliary and the RS clusters of LS are highlighted in blue and red, respectively.

LS belongs to the radical *S*-adenosylmethionine (SAM) superfamily of enzymes, which use a [4Fe–4S] cluster cofactor to cleave SAM reductively to produce a 5′-deoxyadenosyl 5′-radical (5′-dA·).^24−33^ In LS catalysis, the 5′-dA· is used to abstract hydrogen atoms (H·) from C6, and then from C8, of the octanoyllysyl residue to allow for sulfur attachment.^34−37^ Unlike most radical SAM (RS) enzymes, which contain only one [4Fe–4S] cluster, LS contains two [4Fe–4S] clusters.^34,38−40^ One cluster, termed the RS cluster, is found in all RS enzymes and is ligated by three cysteine residues in a conserved CxxxCxxC motif. SAM binds to the unligated iron ion of this cluster, which is a prerequisite for its reductive cleavage.^27,41−43^ The second cluster, termed the auxiliary cluster, is bound by cysteines in an N-terminal CxxxxCxxxxxC motif and a C-terminal serine in a conserved RSSY motif (Figure 2). This cluster has been shown to be the source of the appended sulfur atoms, resulting in its degradation as a result of turnover.^36,44,45^ Therefore, LS catalyzes no more than 1 turnover during in vitro reactions in the absence of a system that restores the auxiliary [4Fe–4S] cluster.^46,47^

Recently, it was shown that the iron–sulfur (Fe–S) cluster carrier protein *E. coli* NfuA can efficiently regenerate the auxiliary [4Fe–4S] cluster of *E. coli* LipA, permitting LipA to perform multiple turnovers.^46,47^ Fe–S cluster assembly and repair is a highly regulated process that is coordinated by a complex network of proteins. In bacteria, yeast, and human model systems, de novo biogenesis of Fe–S clusters involves transient assembly of clusters on the scaffold protein ISCU (IscU in bacteria), with their subsequent transfer directly to recipient apo acceptor proteins or to a subset of late-acting carrier proteins.^48−55^ Among others, mitochondrial proteins BOLA3, ISCA1, ISCA2, GLRX5, and NFU1 have been implicated in Fe–S cluster assembly and trafficking in humans.^52,56−58^ Select mutations in genes encoding these proteins have been reported to be pathogenic and to cause severe infantile disorders of systemic energy metabolism and multiple mitochondrial dysfunctions syndrome types 1–5 (MMDS).^48,59,60^ Some of the severe manifestations of MMDS include weakness, respiratory failure, lack of neurologic development, hyperglycemia, lactic acidosis, and early death,^61−64^ symptoms associated with defective lipoic acid biosynthesis. Biochemical features of patients with pathogenic mutations in genes encoding these proteins include decreased activities of several mitochondrial Fe–S cluster-containing enzymes, including complex-I, complex-II, and LIAS.^65^ These studies suggest that BOLA3, ISCA1, ISCA2, GLRX5, and NFU1 may play important roles in Fe–S cluster biogenesis, trafficking, and regeneration mechanisms, especially for LIAS. However, exactly how these proteins function remains elusive.

To date, our understanding of the mechanism of lipoyl cofactor formation is derived mainly from in vivo and in vitro studies of the *E. coli* enzyme,^10,24,34,36,37,39,66−69^ with additional insight from studies of enzymes from *Sulfolobus solfataricus*,^70^*Thermosynechococcus elongatus*,^44^ and *Mycobacterium tuberculosis*.^71,72^ Only recently has human LIAS been isolated and investigated in vitro. In one study, LIAS was used to show how paramagnetic NMR can be leveraged to demonstrate SAM and substrate binding to RS enzymes.^73^ In a second study, the effect of ISCA2 and ISCU [among other iron sulfur cluster (ISC) assembly proteins] on the de novo in vitro reconstitution of the Fe–S clusters on LIAS was assessed.^74^ However, neither study determined LIAS activity quantitatively nor specifically addressed how the auxiliary cluster of LIAS is restored during turnover. The lack of robust in vitro biochemical studies on LIAS, and particularly how the implicated Fe–S cluster trafficking proteins BOLA3, ISCA1, ISCA2, GLRX5, and NFUI function in lipoyl cofactor biosynthesis, led us to investigate how the auxiliary cluster of LIAS is regenerated after turnover.

Herein, we show that NFU1 and LIAS form a tight complex in vitro, as has been shown in in vivo yeast two-hybrid studies,^75^ and that NFU1 can efficiently restore the auxiliary cluster of LIAS during turnover. We also investigate several additional Fe–S cluster carrier proteins, including BOLA3, previously identified as being critical in the biosynthesis of the lipoyl cofactor in humans and *Saccharomyces cerevisiae*. However, our in vitro studies suggest that BOLA3 has no direct effect on Fe–S cluster transfer from NFU1 or GLRX5 to LIAS. Further, we show that ISCA1 and ISCA2 can enhance LIAS turnover, but only slightly.

## Results

### Isolation and Characterization of LIAS

**Figure 3:**
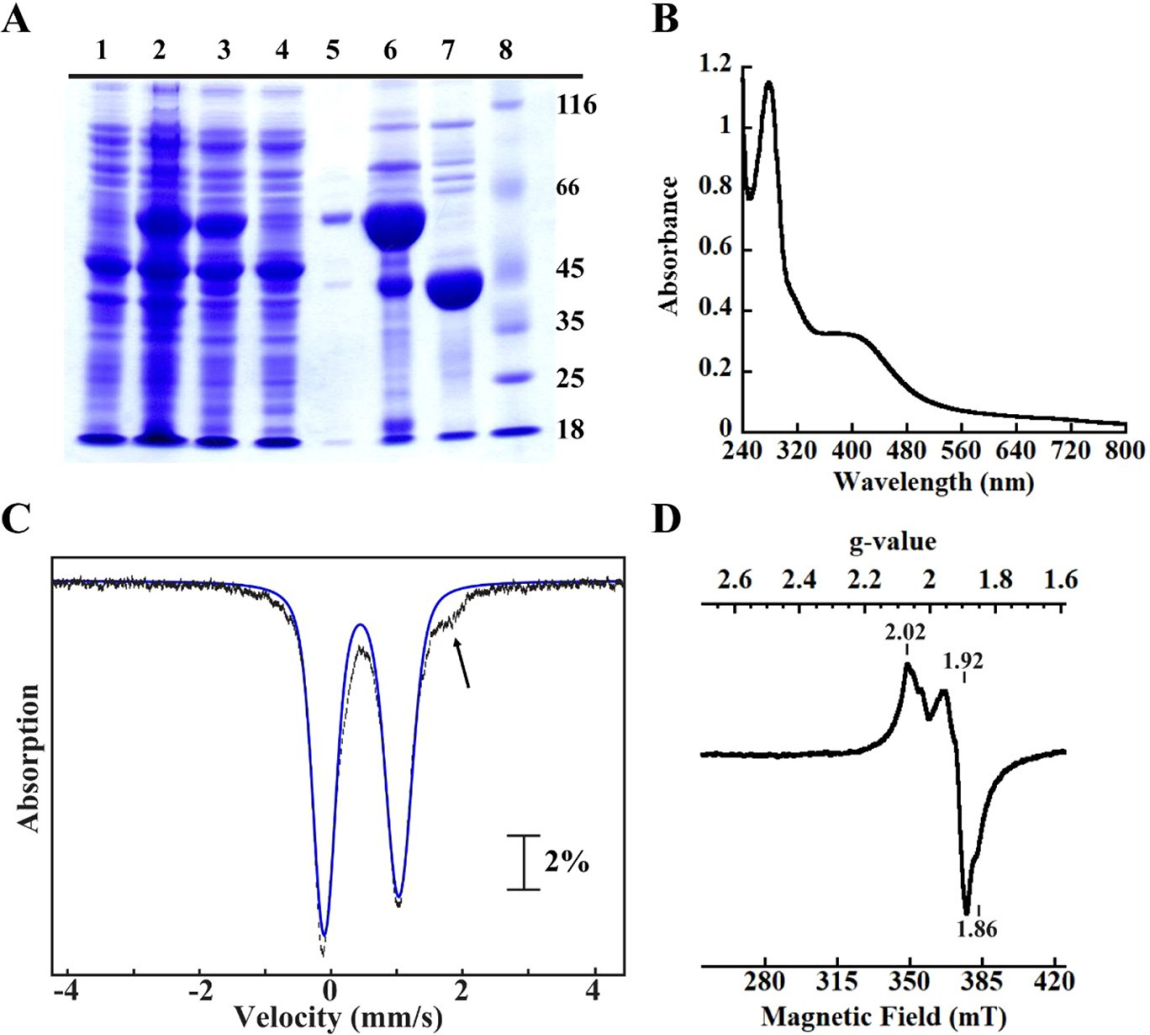
Biophysical characterization of LIAS: SDS–PAGE gel analysis of the expression and purification of LIAS on a Ni–NTA column (A), UV–vis scan of LIAS (B), Mössbauer spectrum of LIAS (C), and EPR spectrum of dithionite-reduced LIAS (D). (A) Lane 1, pre-IPTG induction; lane 2, post-IPTG induction; lane 3, crude lysate; lane 4, Ni–NTA column flow-through; lane 5, wash; lane 6; SUMO–LIAS fusion eluate; lane 7, LIAS after the SUMO tag is removed; lane 8, protein molecular weight ladder. (B) The UV–vis absorption scan of 8 μM purified LIAS showing a broad absorption at ∼410 nm, which is typical for proteins that bind [4Fe–4S] clusters. (C) The Mössbauer spectrum of 380 μM LIAS at 4.2 K, collected in the presence of a 53 mT external magnetic field applied parallel to the direction of propagation of the γ beam. The vertical bars represent the experimental spectrum, and the blue line shows the features associated with a [4Fe–4S]^2+^ cluster. The arrow indicates the shoulder resulting from spectral features of a site-differentiated [4Fe–4S]^2+^ cluster. (D) The EPR spectrum of 400 μM LIAS reduced with 4 mM dithionite and collected at 10 K with 10 mW microwave power and 0.2 mT modulation amplitude confirming bound [4Fe–4S] clusters.

Full-length human LIAS (UniProtKB O43766) is composed of 372 amino acids; however, there are several predicted isoforms of shorter length. Importantly, the first 27 amino acids are predicted to form the mitochondrial targeting sequence. When we attempted to express this full-length construct, almost all the protein was produced in inclusion bodies. Similar to two recent publications,^73,74^ we therefore engineered a construct (amino acids 28–372) that lacked the mitochondrial targeting sequence, which expressed well in *E. coli* and was amenable to purification with its two [4Fe–4S] cluster cofactors largely intact. This protein contained an N-terminal SUMO tag, which was removed after purification, affording an LIAS variant containing a Gly-His N-terminal appendage. The *LIAS* gene was expressed along with genes on plasmid pDB1282, which harbors the *Azotobacter vinelandiiisc* operon.^76^ This coexpression strategy has been shown to enhance the production of soluble *E. coli* LipA as well as its Fe–S cluster content.^34^ The protein was purified under anoxic conditions using Ni–NTA immobilized metal affinity chromatography (Ni–IMAC) followed by size-exclusion chromatography (SEC) to remove impurities such as protein aggregates and unbound iron and sulfide species. Upon purification, the protein was found to be ≥95% homogeneous (Figure 3A) and contained 9.5 ± 0.1 iron and 5.5 ± 0.1 sulfide ions per polypeptide after accounting for a correction factor of 1.6 for the Bradford method of protein quantification, which was established by quantitative amino acid analysis. The UV–vis spectrum (Figure 3B) showed a broad absorption feature at ∼410 nm typical of [4Fe–4S] cluster binding proteins. The 4.2 K/53 mT Mössbauer spectrum of LIAS (Figure 3C) is dominated by a quadrupole doublet with parameters [isomer shift (δ) = 0.46 mm/s, quadrupole splitting (Δ*E*_Q_) = 1.14 mm/s; ∼87% of total intensity, 1.7 equiv of [4Fe–4S] per LIAS, blue line] typical of [4Fe–4S]^2+^ clusters.^77^ The shoulder indicated by the arrow is at a position typically observed for the “Fe(II)-like” site of a site-differentiated [4Fe–4S]^2+^ cluster.^36^ Analysis of the field dependence of spectra collected in a 53 mT magnetic field and zero field (Figure S1) suggests the presence of ∼0.3 equiv of [3Fe–4S]^0^ cluster. The electron paramagnetic resonance (EPR) spectrum of purified LIAS after reduction with dithionite and recorded at 10 K (Figure 3D) shows a rhombic signal typical of [4Fe–4S]^+^ clusters, with estimated *g*-values of 2.02, 1.92, and 1.86. The UV–vis, Mössbauer, and EPR spectra in concert with iron and sulfide quantification are consistent with the presence of two [4Fe–4S]^2+^ clusters, as has been shown previously for human LIAS^74^ and the *E. coli*,^34^*M. tuberculosis*,^71,72^ and *T. elongatus* enzymes.^44^ It should be noted that our iron quantification shows 1.5 irons beyond the eight that our model suggests and that this additional iron is not observed in the Mössbauer spectrum. We believe that the discrepancy is due to systematic inaccuracies in iron and protein quantification.

### Isolation and Characterization of NFU1

**Figure 4:**
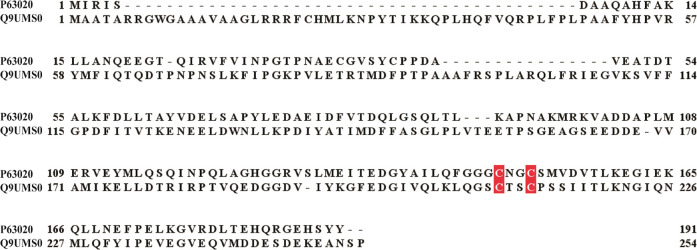
Amino acid sequence alignment of *E. coli* NfuA (P63020) and human NFU1 (Q9UMS0) showing the two conserved cysteine residues that ligate the Fe–S cluster highlighted in red.

In a recent in vitro study, *E. coli* NfuA was shown to regenerate the auxiliary cluster of *E. coli* LipA after each turnover, thus allowing LipA to act catalytically.^47^ There have also been several recent in vitro studies of NFU1, the human ortholog of *E. coli* NfuA. In one study, focused on NMR solution structures of NFU1, it was shown that holo-NFU1 could donate its [4Fe–4S] cluster to apo aconitase,^78^ while in another, holo-NFU1 was reported to transfer a [2Fe–2S] cluster to apo ferredoxin 1 and ferredoxin 2.^79^ Given the effects of *E. coli* NfuA on *E. coli* LipA turnover and the activation of aconitase by NFU1, we assessed the effect of NFU1 on LIAS turnover. Like NfuA, NFU1 is bimodular, with a degenerate N-terminal A-type domain and a highly conserved NifU-like C-terminal Fe–S cluster binding domain. Two cysteine residues in a conserved C-terminal CxxC motif are believed to serve as ligands to a [4Fe–4S] formed at the interface of two monomers both in NfuA and in NFU1 (Figure 4), although there have been disagreements in the literature concerning whether this form is biochemically relevant or whether a form containing a [2Fe–2S] cluster is more relevant.^64,80^

**Figure 5:**
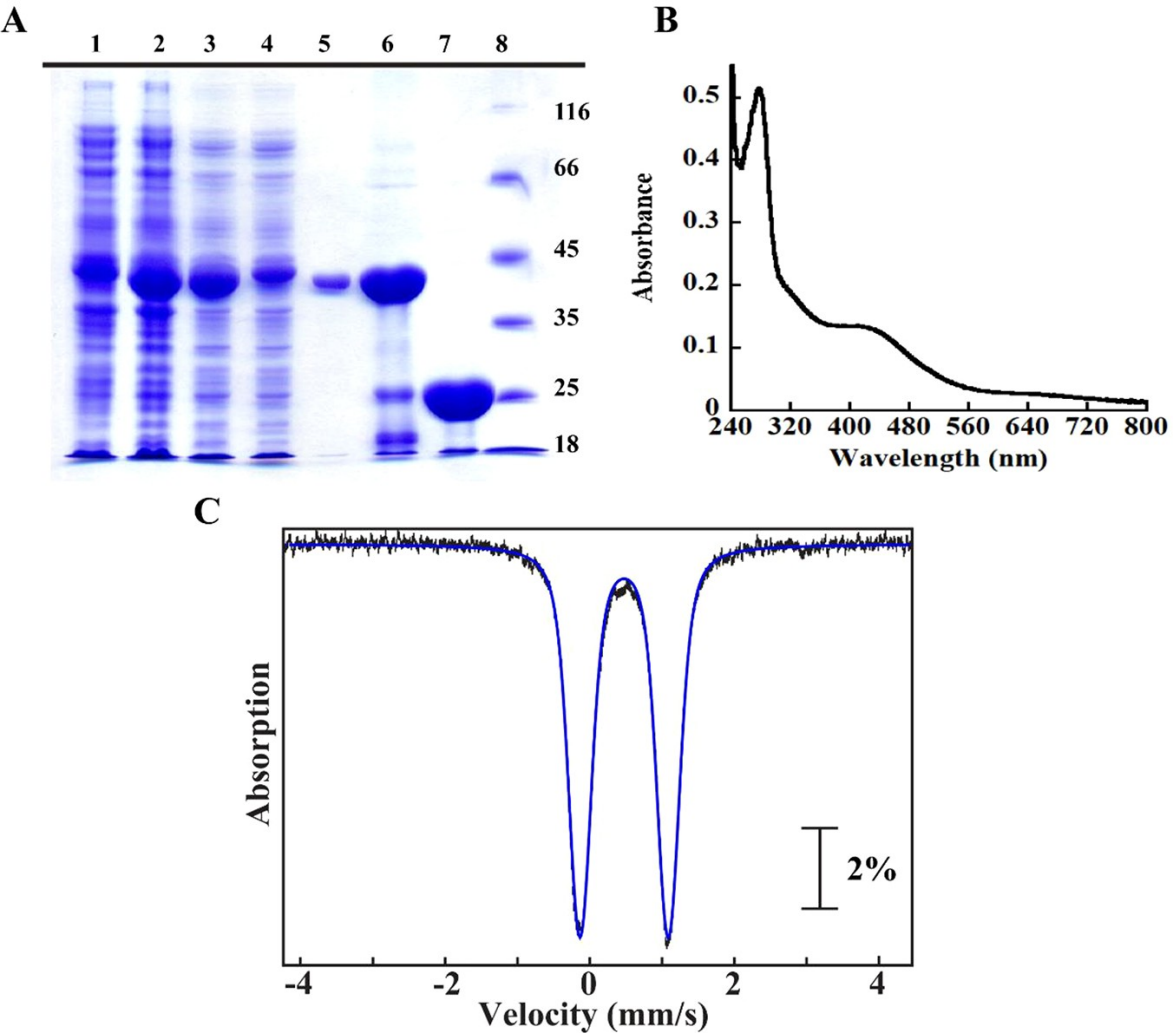
SDS–PAGE analysis of the expression and purification of NFU1 on a Ni–NTA column (A), UV–vis scan of 15 μM NFU1 (B), and Mössbauer spectra of NFU1 (C). (A) Lane 1, pre-IPTG induction; lane 2, post-IPTG induction; lane 3, crude lysate; lane 4, Ni–NTA column flow-through; lane 5, wash; lane 6, SUMO–NFU1 fusion eluate; lane 7, NFU1 after the SUMO tag is removed; lane 8, protein molecular weight ladder. (B) The UV–vis absorption scan spectrum of 15 μM purified NFU1 showing a broad absorption at ∼410 nm indicative of a bound [4Fe–4S] cluster. (C) The 4.2 K Mössbauer spectra of 860 μM NFU1 in the presence of a 53 mT external magnetic field applied parallel to the direction of propagation of the γ beam. The vertical bars represent the experimental spectra, and the blue line shows the features associated with a [4Fe–4S]^2+^ cluster.

To study the effect of NFU1 on LIAS activity, we first expressed and purified the full-length construct (amino acids 1–254) and a construct lacking the mitochondrial transit sequence (amino acids 10–254). Unfortunately, both constructs led to poorly behaved protein products that existed mostly in higher order oligomeric states that were difficult to resolve by SEC. We therefore tried a shorter N-terminally truncated variant (amino acids 59–254). The region that was removed is predicted by AlphaFold^81^ (AF-Q9UMS0-F1) to be highly unstructured, while the truncated variant is well-structured and has been reported in previous studies.^78,82^ The construct was overproduced in the presence of plasmid pDB1282 and expressed as a fusion with an N-terminal SUMO tag that was removed during purification to yield NFU1 containing a Gly-His N-terminal appendage. The protein was isolated under anoxic conditions using Ni–IMAC followed by SEC and shown to be ≥95% homogeneous by sodium dodecyl sulfate–polyacrylamide gel electrophoresis (SDS–PAGE) gel analysis (Figure 5A). The UV–vis spectrum of the protein, with a distinctive feature at 410 nm, is consistent with the presence of a [4Fe–4S] cluster (Figure 5B), as has been observed in previous studies.^75^ Analysis of ^57^Fe-labeled NFU1 by Mössbauer spectroscopy (Figure 5C) reveals that the spectrum is dominated by a single quadrupole doublet (δ = 0.48 mm/s, Δ*E*_Q_ = 1.20 mm/s; ∼97% of total intensity, blue line) typical of [4Fe–4S]^2+^ clusters.^77^ When NFU1 was analyzed by EPR, a very weak signal was observed with and without dithionite reduction (Figure S2), suggesting that the [4Fe–4S] cluster is not easily reduced to the [4Fe–4S]^+^ state. Iron and sulfide analysis indicated that the isolated protein contained 2.40 ± 0.02 irons and 3.00 ± 0.03 sulfides per polypeptide, consistent with a bridging [4Fe–4S] cluster between two NFU1 monomers.

### NFU1 Binds Tightly to LIAS

**Figure 6:**
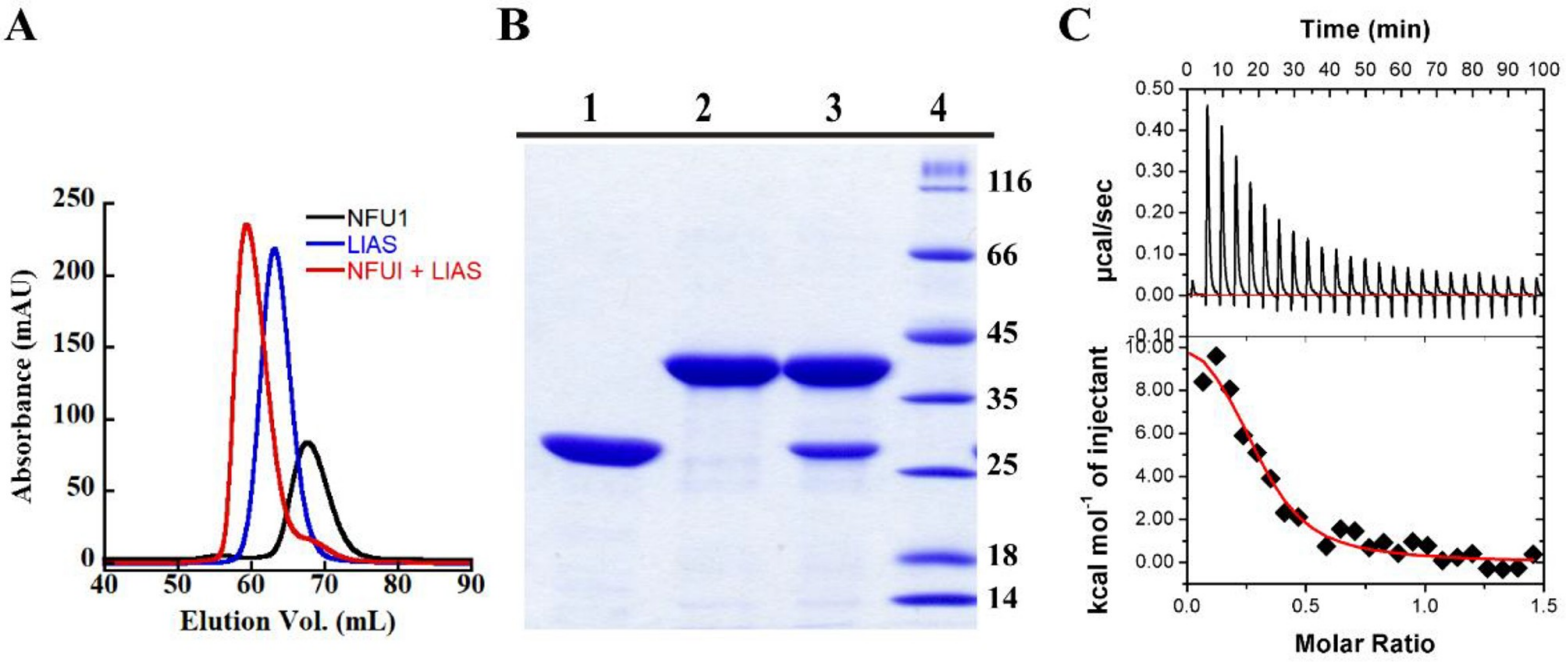
Size-exclusion gel filtration chromatography elution profiles of holo-LIAS (100 μM, blue), holo-NFU1 (200 μM, black), and a 1:1 mixture of holo-LIAS and holo-NFU1 (100 μM each, red) (A). SDS–PAGE analysis of the chromatographed proteins: lane 1, holo-NFU1 alone; lane 2, holo-LIAS alone; lane 3, a mixture of holo-NFU1 and holo-LIAS, indicating complex formation (B). ITC binding results of LIAS titrated into NFU1, showing entropically driven binding with a dissociation constant (*K*_D_) of 0.7 ± 0.2 μM (C).

Our previous studies indicated that *E. coli* NfuA binds tightly to *E. coli* LipA, suggesting that NFU1 might interact similarly with LIAS, as has been determined recently through yeast two-hybrid analysis.^75^ To assess whether NFU1 and LIAS form a tight complex in vitro, the holo (Fe–S cluster containing) forms of the two proteins were analyzed separately and together by SEC (Figure 6A). LIAS alone (blue trace) elutes at 62.5 mL, exhibiting an experimentally calculated mass of 46 kDa (theoretical mass, 39.2 kDa) based on the elution profiles of a suite of standards. NFU1 alone (black trace) elutes at 67.7 mL, exhibiting an experimentally calculated mass of 29.8 kDa (theoretical mass, 22 kDa). The sample containing both LIAS and NFU1 (red trace) shows an elution volume of 59.1 mL, corresponding to an experimentally calculated mass of 61.2 kDa, suggestive of a 1:1 heterodimer of LIAS and NFU1 (theoretical mass, 61.2 kDa). To confirm the results obtained by SEC, we subjected fractions from the peaks observed in the LIAS alone, NFU1 alone, and LIAS plus NFU1 traces to SDS–PAGE (Figure 6B). As shown, the peak from the LIAS plus NFU1 sample (lane 3) contains both NFU1 and LIAS. In these experiments, NFU1 migrates as a monomer and interacts with LIAS as a monomer. We further characterized the interactions between NFU1 and LIAS by determining the equilibrium binding dissociation constant (*K*_D_) using isothermal titration calorimetry (ITC, Figure 6C). The results from ITC indicate a *K*_D_ value of 0.7 ± 0.2 μM, confirming the strong interaction.

### In Vitro Determination of LIAS Activity

**Figure 7:**
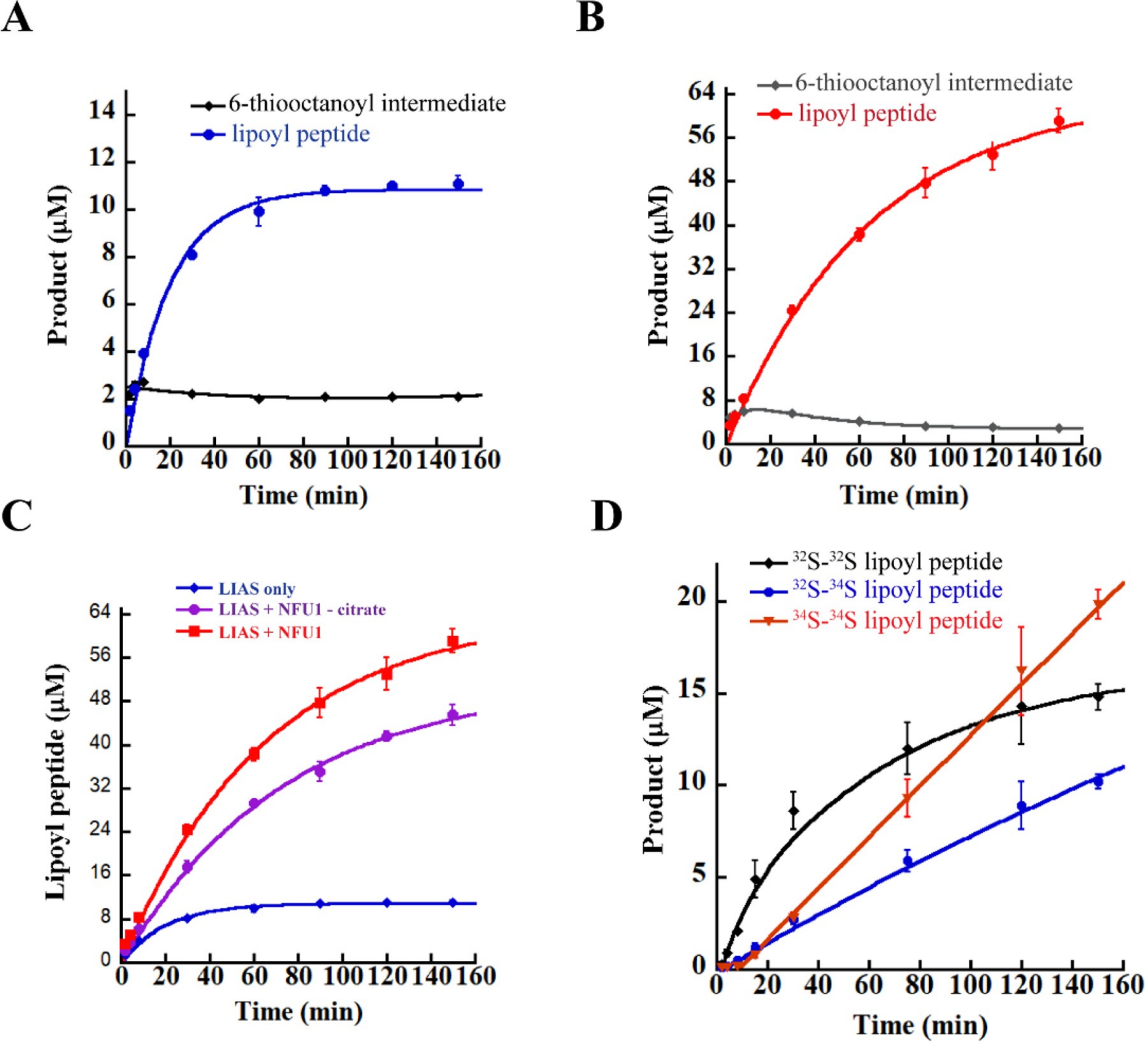
LIAS activity determinations: LIAS (10 μM) activity in the absence of NFU1 (A), in the presence of 200 μM NFU1 (B), in the presence of both 200 μM NFU1 and 5 mM sodium citrate (C), and in the presence of 200 μM NFU1 reconstituted with ^34^S-labeled sulfide (D). LIAS alone catalyzes about 1 turnover of lipoyl product (blue trace) with the 6-thiooctanoyl intermediate quickly reaching a steady level (black trace) (A). The inclusion of an excess of NFU1 in the LIAS reaction promotes multiple turnovers and generation of more than 5 equiv of lipoyl product (red trace), while the formation and decay of the intermediate mimics that of LIAS alone (gray trace) (B). The inclusion of 5 mM sodium citrate, a divalent metal chelator, does not significantly alter the effect of NFU1 (purple trace) compared to reactions in which citrate is omitted (red trace) (C). In the presence of NFU1 reconstituted with ^34^S^2–^, the ^32^S-labeled lipoyl peptide product is formed first before formation of the mixed ^32^S–^34^S (blue trace) and ^34^S–^34^S-labeled (red trace) lipoyl peptide products (D). The data in panels C and D suggest direct cluster transfer from NFU1 to LIAS during turnover. Unless otherwise noted, all activity assays included the following at their indicated final concentrations: 350 μM octanoyl peptide substrate, 0.75 mM SAM, and 10 μM SAH nucleosidase. The reactions were carried out at room temperature in a buffer that contained 50 mM HEPES, pH 7.5, and 0.25 M KCl and were initiated with a final concentration of 1 mM dithionite. The respective data shown in panels A–D are averages from assays done in triplicate, and the error bars represent one standard deviation from the mean. The 6-thiooctanoyl intermediate data were fit to an exponential equation that accounts for its formation and decay phases (A and B), while the lipoyl peptide product data were fit to a biphasic double-exponential rate of formation equation, assuming an A → B → C model, as has been previously reported for *M. tuberculosis* LipA (ref (72)).

LIAS in vitro activity was determined in assays using a four amino acid peptide substrate mimic containing an octanoyllysyl residue [Glu-(N^6^-octanoyl)Lys-Ala-Tyr], a shorter version of the eight amino acid peptide previously used in *E. coli* LipA^36,47^ and *M. tuberculosis* LipA^72^ assays. For reasons that we do not understand, this shorter 4-mer peptide affords more turnovers than the longer 8-mer peptide. As shown in Figure 7A, the protein catalyzes formation of no more than 1 equiv of lipoyl product (blue trace), with no significant accumulation of the 6-thiooctanoyl intermediate (black trace), consistent with previous studies of *E. coli* LipA that indicate that the auxiliary cluster gets degraded as a function of turnover and that both sulfurs are contributed by the same LIAS polypeptide.^68^

### In Vitro Determination of NFU1 Effect on LIAS Activity

After establishing that NFU1 binds tightly to LIAS, we assessed whether NFU1 affects LIAS activity. As shown in Figure 7A, LIAS produces not more than 1 equiv of product in the absence of NFU1. By contrast, in the presence of excess NFU1 (200 μM monomer), 10 μM LIAS catalyzes more than 5 turnovers in a time-dependent manner over 150 min (Figure 7B, red trace). The lack of a clear burst followed by a slower phase of product formation suggests that regeneration of LIAS’s auxiliary cluster is not rate-limiting, similar to what was observed with *E. coli* LipA and NfuA.^47^ The amount of observed lipoyl product is less than what would be expected, given that the NFU1 dimer is in a 10-fold excess. Currently, we attribute the leveling off of activity to aberrant chemistry during destruction of the auxiliary cluster and its subsequent reconstitution. We predict that a more physiological system, especially with respect to the choice of reductant, may enhance the extent of turnover. To confirm that cluster transfer from NFU1 to LIAS is largely direct rather than a result of release of iron and sulfide into solution, the effect of NFU1 on LIAS activity was probed in the presence of 5 mM sodium citrate. Under these conditions, citrate will sequester free iron liberated by NFU1 and prevent it from being used to reconstitute LIAS. The observation that the presence of 5 mM citrate shows no dramatic effect on overall lipoyl product formation (Figure 7C) suggests that the transfer is direct, consistent with the finding that NFU1 and LIAS form a complex. The somewhat reduced activity is attributed to the ability of citrate to remove the cluster from NFU1, as was observed previously in experiments conducted with *E. coli* IscU.^47^ Direct cluster transfer was further assessed by performing LIAS activity assays in the presence of an NFU1 protein that was chemically reconstituted with ^34^S^2–^ to yield NFU1 containing a [4Fe–4^34^S] cluster. Results from these studies show relatively rapid formation of ^32^S–^32^S-containing lipoyl product, which is followed by slower production of ^32^S–^34^S-containing and ^34^S–^34^S-containing lipoyl product (Figure 7D). The formation of lipoyl product containing the mixed isotope (^32^S–^34^S) was also observed with the *E. coli* NfuA/LipA system, although larger amounts of this product are formed with NFU1/LIAS.^47^ It is not clear whether mixed-isotope formation is due to the conditions of the assay (e.g., presence of dithionite or incomplete LIAS reconstitution) or is intrinsic to how the cluster is transferred from NFU1/NfuA to LIAS/LipA. In reactions containing NfuA and LipA, almost 2 equiv of ^32^S–^32^S-containing lipoyl product was formed before formation of the ^34^S-containing species. In the NFU1/LIAS reactions herein, it appears that almost 1.5 equiv of the ^32^S–^32^S-containing lipoyl product is formed during the reaction. These data suggest that potentially all four sulfides of the auxiliary cluster of LIAS can be used for lipoyl product formation. It should be noted that the total amount of lipoyl product generated when using ^34^S-reconstituted NFU1 is lower than that when using NFU1 at natural abundance. We believe the difference is related to how each protein is produced. To generate ^34^S-reconstituted NFU1, the protein is first isolated in its apo form before being reconstituted. By contrast, NFU1 at natural abundance is produced directly in its Fe–S cluster form, and then further reconstituted.

### In Vitro Effect of NFU1 plus BOLA3 on LIAS Activity

Previous studies have implicated BOLA3 in lipoyl cofactor formation; however, it has not been established when exactly BOLA3 functions. BOLA3 is a mitochondrial Fe–S cluster assembly and trafficking factor that facilitates Fe–S cluster insertion into a subset of Fe–S cluster acceptor proteins, and it has been suggested that it might act synergistically with NFU1.^84^ BOLA3 was previously reported to form heterodimeric complexes with NFU1 and also GLRX5, and its deletion was associated with defective lipoic acid biosynthesis in vivo, suggesting its requirement for LIAS Fe–S cluster maturation.^65,84^ With this precedent in mind, we tested whether BOLA3 could act synergistically with NFU1 for Fe–S cluster transfer to LIAS during turnover. However, inclusion of BOLA3 in our LIAS activity assays in the presence of NFU1 does not show any additional effect on turnover (Table 1 and Figure S3A). Moreover, SEC experiments similar to those conducted with LIAS and NFU1 show that BOLA3 does not bind tightly to LIAS.

**Table 1:** Effect of Fe–S Cluster Assembly Proteins on LIAS Activity^a^

NFU1	BOLA3	ISCA1	ISCA2	ISCU	GLRX5	effect on LIAS activity^b^
X						increase to 5–6 turnovers
X	X					same as NFU1 alone
		X				increase to ∼1.5 turnovers
			X			increase to ∼2 turnovers
				X		slight increase to ∼1 turnover
					X	no observed effect
	X				X	no observed effect

a X denotes the presence of that protein in reaction mixtures.

b Due to differences in LIAS cluster content from batch to batch, the observed turnover numbers vary by about 10–20%. We present the best turnover numbers observed for each corresponding protein or protein mixture.

### In Vitro LIAS Activity Determination in the Presence of GLRX5, ISCA1, ISCA2, and ISCU

ISCA2 and ISCU have also been reported to be able to reconstitute LIAS, transforming an inactive protein into one that is competent for catalysis.^74^ In other work, *E. coli* IscU was reported to reconstitute *E. coli* LipA, allowing for multiple turnovers,^47^ while in another study, ISCA1, ISCA2, and IBA57 have been implicated in late-stage [4Fe–4S] cluster maturation, including Fe–S clusters in LIAS.^85^ On the basis of these studies, we explored the effect of including purified and appropriately reconstituted GLRX5, ISCA1, ISCA2, and ISCU proteins in LIAS activity assays. Additionally, the effect of BOLA3 in activity assays that also included GLRX5 was tested. In summary, when either ISCA1 or ISCA2 are included in LIAS activity assays, only a modest increase in turnover is observed, up to an additional ∼0.5 and ∼1 equiv for ISCA1 and ISCA2, respectively. No change in LIAS turnover is observed by inclusion of ISCU, GLRX5, or BOLA3 in the presence of GLRX5 (Table 1 and Figure S3, parts B and C).

## Discussion

LS reductively cleaves SAM to generate 5′-dA· that abstracts substrate hydrogen atoms to allow for sequential sulfur insertions to produce the lipoyl cofactor. During turnover, LS sacrifices its auxiliary [4Fe–4S] cluster as the source of the sulfur atoms incorporated into the product, a process that leads to degradation of the auxiliary cluster. Thus, the enzyme is rendered inactive after a single turnover in the absence of a system that can regenerate the auxiliary cluster either by repairing it or by fully replacing it. Fe–S clusters are essential in all domains of life and are involved in various cellular processes, including respiration, ribosome assembly, DNA repair, and the biosynthesis of key metabolites.^86−89^ Due to the complexity of ISC assembly, trafficking, and repair mechanisms in vivo, these processes are yet to be fully understood. Understanding these mechanisms in humans is vital because Fe–S clusters and their incorporation into proteins that require them are critical to many serious diseases.^55,56,63,90−92^ While recent in vitro attempts to characterize Fe–S cluster assembly and trafficking have been reported, the determination of which late-acting carriers mediate LIAS auxiliary cluster regeneration during catalytic turnover remains unresolved. Here, we have investigated the effects of select human mitochondrial Fe–S cluster carriers (BOLA3, ISCA1, ISCA2, ISCU, GLRX5, and NFU1) on LIAS auxiliary cluster reconstitution during turnover and demonstrate for the first time that, like its *E. coli* counterpart NfuA, human NFU1 can efficiently reconstitute human LIAS during turnover to promote catalytic activity. Further, we show that NFU1 interacts tightly with LIAS, corroborating a similar finding from previously reported yeast two-hybrid experiments.^75^ By contrast, ISCA1 and ISCA2 only partially enhance turnover of LIAS. It is likely that ISCA1 and ISCA2 are involved in de novo Fe–S incorporation into LIAS and not incorporation during catalytic turnover. Moreover, our studies suggest that neither BOLA3, GLRX5, nor ISCU are involved in the immediate regeneration of the auxiliary cluster of LIAS during turnover, although it appears that ISCU, like ISCA1 and ISCA2, can reconstitute LIAS de novo. Our studies suggest that the mechanism by which NFU1 transfers its cluster to LIAS during turnover is distinct from de novo reconstitution of LIAS and provide the basis for an additional system on which to investigate this transfer.

## Materials and Methods

### Materials

*N*-(2-Hydroxyethyl)-piperazine-*N*′-(2-ethanesulfonic acid) (HEPES) was purchased from Fisher Scientific. Imidazole was purchased from J. T. Baker Chemical Co. Potassium chloride and glycerol were purchased from EMD Chemicals. 2-Mercaptoethanol (BME), sodium dithionite, phenylmethylsulfonyl fluoride (PMSF), pyridoxal 5′-phosphate (PLP), and sodium sulfide were purchased from MilliporeSigma. Dithiothreitol (DTT), tris(2-carboxyethyl)phosphine hydrochloride (TCEP-HCl), kanamycin, ampicillin, arabinose, and isopropyl β-d-1-thiogalactopyranoside (IPTG) were purchased from Gold Biotechnology. Nickel-nitrilotriacetic acid (Ni-NTA) resin was acquired from Qiagen. *S*-Adenosyl-l-methionine (SAM) was synthesized and purified as described previously.^93^ Restriction enzymes and materials for cloning were obtained from New England Biolabs (Ipswich, MA). DNA isolation kits were purchased from Macherey-Nagel (Dueren, Germany). All other chemicals and materials were of the highest grade available and were from MilliporeSigma.

All peptides used in this study were custom-synthesized by Proimmune (Oxford, U.K.) except the octanoyl-containing peptide substrate [Glu-(N^6^-octanoyl)Lys-Ala-Tyr], which was synthesized by Genscript (Piscataway, NJ, U.S.A.). AtsA peptide (Pro-Met-Ser-Ala-Pro-Ala-Arg-Ser-Met) was used as an external standard for quantification of peptide products during liquid chromatography–mass spectrometry (LC–MS) analysis. An 8-thiooctanoyl-containing peptide [Glu-(N^6^-8-thiooctanoyl)Lys-Ala-Tyr] and a lipoyl-containing peptide [Glu-(N^6^-lipoyl)Lys-Ala-Tyr] were used as product standards for the 6-thiooctanoyl intermediate and lipoyl products, respectively.

### General Methods

UV–vis spectra were recorded on a Varian Cary 50 spectrometer (Walnut Creek, CA) using the WinUV software package to control the instrument. Ultraperformance liquid chromatography (UPLC) was conducted on an Agilent Technologies 1290 Infinity II system coupled to an Agilent Technologies 6470 QQQ mass spectrometer (Santa Clara, CA) with detection by tandem mass spectrometry (MS/MS). Data collection and analysis were performed using the associated MassHunter software package.

### Plasmids and Strains

Genes encoding *Homo sapiens* BOLA3 (aa 27–107), ISCA1 (aa 13–129), ISCA2 (aa 9–154), ISCU (aa 35–167), GLRX5 (aa 32–157), LIAS (aa 28–372), and NFU1 (aa 59–254) without their respective mitochondrial targeting sequences were synthesized at ThermoFisher Scientific after codon optimization using GeneArt software (ThermoFisher Scientific) for protein overexpression in *E. coli*. The genes were subcloned into a modified pSUMO plasmid (LifeSensors Inc.), (pDWSUMO), and pET28a vectors using *Nde*I and either *Xho*I or *Bam*HI restriction sites. After sequence verification by DNA sequencing at the Penn State Genomics Core Facility (University Park, PA), the resulting plasmids, pDWSUMO-BOLA3, pDWSUMO-ISCA1, pDWSUMO-ISCA2, pDWSUMO-ISCU, pDWSUMO-GLRX5, pDWSUMO-LIAS, and pDWSUMO-NFUI (and their corresponding pET28a counterparts), were separately used to transform *E. coli* BL21 (DE3) competent cells containing the pDB1282 plasmid, which harbors the *isc* operon from *A. vinelandii*.^94^ As cloned, each of the proteins in the pDWSUMO plasmid was expressed as a fusion with an N-terminal SUMO tag that also contained a His_6_-tag at the N-terminus, while their counterparts cloned in pET28a were expressed with an N-terminal His_6_-tag. During purification, the SUMO tag was removed using ULP1 protease, affording corresponding pure proteins with a Gly-His appendage at the N-terminus.

### Growth and Expression of BOLA3, ISCA1, ISCA2, ISCU, GLRX5, LIAS, and NFU1

All proteins were overexpressed in *E. coli* using the following general procedure with minor adjustments for BOLA3 since it is not an Fe–S carrier protein by itself. In a typical growth, a 200 mL starter culture containing 25 μg/mL kanamycin (50 μg/mL for BOLA3) and 50 μg/mL ampicillin (no ampicillin was included for BOLA3) was inoculated with a single colony and incubated overnight at 37 °C with shaking at 250 rpm. A 25 mL aliquot of the starter culture was used to inoculate 4 L of M9 minimal medium containing appropriate antibiotic(s) as above and incubated at 37 °C with shaking (180 rpm) until an optical density at 600 nm (OD_600_) of 0.3 was reached. At OD_600_ 0.3, 0.2% (w/v) l-arabinose was added to induce the expression of genes on the pDB1282 plasmid (except for BOLA3). At OD_600_ ∼ 0.6, 50 μM FeCl_3_ and 100 μM l-cysteine were added (except for BOLA3), and the culture was cooled in ice–water for 1 h with occasional shaking. Protein expression was induced by adding IPTG to a concentration of 0.2 mM, and incubation was continued at 18 °C with shaking at 180 rpm for an additional 12 h. The cells were harvested at 4 °C by centrifugation at 6000*g* for 15 min, flash-frozen in liquid nitrogen, and stored under liquid nitrogen until needed.

### Protein Purification of BOLA3, ISCA1, ISCA2, ISCU, GLRX5, LIAS, and NFU1

The purification of each of the proteins was performed in an anaerobic chamber containing <1 ppm of O_2_ (Coy Laboratory products, Grass Lake, MI) by IMAC using the following general procedures with minor modifications for BOLA3. Cells were resuspended in 200 mL of lysis buffer (100 mM Tris–HCl, pH 8.0, 150 mM KCl, 10 mM imidazole, 10 mM BME, 10 mM MgCl_2_). To the resuspended cells, the following were added at their indicated final concentrations: 0.25 mM FeCl_3_, 1 mM l-cysteine, 1 mM PLP, one SIGMAFAST protease inhibitor tablet, 1 mM PMSF, 0.5 mg/mL lysozyme, and 0.01 mg/mL DNase (no FeCl_3_, cysteine, or PLP was added to BOLA3 samples). The cells were disrupted by sonication with an ultrasonic cell disruptor (Branson Sonifier II “model W-250”, Heinemann), and the lysates were clarified by centrifugation (4 °C, 45 000*g*, 1 h). The N-terminally His_6_-tagged SUMO fusion protein was then purified by Ni–NTA affinity chromatography. The Ni–NTA column was pre-equilibrated with 150 mL of lysis buffer. After loading the supernatant onto the Ni–NTA column, the resin was washed with 200 mL of wash buffer (50 mM HEPES, pH 7.5, 300 mM KCl, 30 mM imidazole, 10% glycerol (v/v), and 10 mM BME). Protein elution from the Ni–NTA resin was performed with 100 mL of elution buffer (50 mM HEPES, pH 7.5, 250 mM KCl, 300 mM imidazole, 10% glycerol, and 10 mM BME). The eluted protein was exchanged into cleavage buffer (50 mM HEPES, pH 7.5, 250 mM KCl, 5% glycerol, 40 mM imidazole, and 10 mM BME) using a PD-10 column. ULP1 protease (50 μg/mg of protein to be cleaved) was added to the fusion protein to excise the His_6_-SUMO tag from the protein of interest, and the reaction mixture was incubated on ice overnight. The following day, the protein sample was reloaded onto the Ni–NTA column pre-equilibrated in cleavage buffer, and the protein of interest was collected in the flow-through. The protein was concentrated to ∼2.5 mL and buffer-exchanged into storage buffer (50 mM HEPES, pH 7.5, 250 mM KCl, 30% glycerol, 2.5 mM TCEP, and 10 mM BME) using a PD-10 column (GE Healthcare). For proteins expressed from pET28a plasmid, their purification followed the same steps as described above, omitting the ULP1 cleavage step. When needed, the proteins were purified further by SEC on a HiPrep 16/60 Sephacryl HR S-200 column (Cytiva) equilibrated in gel filtration buffer (50 mM HEPES, pH 7.5, 250 mM KCl, 10% glycerol, 2.5 mM TCEP, and 10 mM BME) at a flow rate of 0.5 mL/min.^94^ The S-200 column was connected to an AKTA protein liquid chromatography system (Cytiva) in an anaerobic chamber. Fractions containing the target protein were identified by UV–vis absorption at 280 nm and were combined, concentrated, and buffer-exchanged into storage buffer using a PD-10 column.

Amino acid analysis for LIAS and NFU1 was performed by the UC Davis Proteomics Core and revealed that the Bradford method overestimates the protein concentration of LIAS by a factor of 1.6 and that of NFU1 by a factor of 1.1. The concentration of the protein was determined by Bradford method using appropriate correction factors as necessary and using bovine serum albumin (fraction V) as the standard.^95^ The purified protein sample was aliquoted, flash-frozen in liquid N_2_, and stored under liquid nitrogen until needed. Protein homogeneity was judged by 12% SDS–PAGE and was determined to be ≥95% pure. Colorimetric iron and sulfide analyses were conducted on the purified protein using the methods of Beinert.^96−98^

### Overexpression and Purification of ^57^Fe-Labeled LIAS and NFU1 and Mössbauer Spectroscopy

To generate ^57^Fe-labeled proteins for analysis by Mössbauer spectroscopy, LIAS and NFUI proteins were overproduced as described above with the exception that they were supplemented with 50 μM ^57^FeCl_3_ instead of 50 μM FeCl_3_. The growth and purification procedures were essentially identical to those described above, with the exception that ^57^FeCl_3_ was also used for reconstitution during the lysis step. ^57^FeCl_3_ was prepared as previously described.^99^

For analysis by Mössbauer spectroscopy, 380 μM ^57^Fe-labeled LIAS or 860 μM NFU1 was loaded into Mössbauer cups and flash-frozen in liquid nitrogen. Mössbauer spectra were recorded on a spectrometer from SEECO (Edina, MN) equipped with a Janis SVT-400 variable-temperature cryostat. Isomer shifts are reported relative to the centroid of the spectrum of α-iron metal at room temperature. The external magnetic field was applied parallel to the direction of propagation of the γ radiation. The Mössbauer spectra were simulated using the WMOSS spectral analysis software from SEECO (www.wmoss.org, SEE Co., Edina, MN).

### Overexpression and Purification of ^34^S-Labeled NFU1

The expression of apo-NFU1 was as reported for *Ec*NfuA.^47^ The protein was purified as described above with the exception that no FeCl_3_ or cysteine was added during the lysis step, as was done for the other proteins. The purified apo protein was chemically reconstituted with FeCl_3_ and Na_2_^34^S in the same manner as was reported for *Ec*NfuA.^47^ The reconstituted NFU1 was centrifuged at 14 000*g* for 10 min to remove aggregates, concentrated to 2.5 mL, and buffer-exchanged into gel filtration buffer using a PD-10 column. The protein was then further purified on an S-200 SEC column as described above. The synthesis of Na_2_^34^S followed the procedures previously reported.^47^

### Electron Paramagnetic Resonance Spectroscopy Analysis of LIAS and NFUI

For analysis by EPR, 400 μM LIAS or NFU1 in storage buffer was prepared at 4 °C inside an anaerobic chamber. After a 15 min reduction with freshly prepared dithionite (4 mM final concentration), the samples were flash-frozen in cryogenic isopentane. Respective protein samples without dithionite were used as controls. Continuous-wave EPR spectra data were collected at 10 K with a microwave power of 10 mW and a modulation amplitude of 0.2 mT on a Magnettech 5000 X-band ESR spectrometer (Bruker) equipped with an ER 4102ST resonator. The temperature was controlled by an ER 4112-HV Oxford Instruments (Concord, MA) variable-temperature helium-151 flow cryostat.

### Interaction between NFU1 and LIAS

#### Size-Exclusion Chromatography

The ability of NFU1 to interact with LIAS was investigated using size-exclusion gel filtration chromatography. To determine the association, 500 μL of each of the following protein samples was applied to a pre-equilibrated (gel filtration buffer) HiPrep 16/60 Sephacryl HR S-200 column (GE Healthcare) housed in a Coy anaerobic glovebox (<1 ppm of O_2_) and chromatographed using a flow rate of 0.5 mL/min. The elution volumes (*V*_e_) of the following protein samples were determined: 100 μM LIAS alone, 200 μM NFU1 alone, and a 1:1 mixture of 200 μM LIAS plus 200 μM NFU1. For the molecular weight standard calibration curve, four individual injections were chromatographed for the standards as follows: a mixture of 250 μL of cytochrome *c* at 2 mg/mL (12.4 kDa) plus 250 μL of β-amylase at 4 mg/mL (200 kDa); a mixture of 250 μL of carbonic anhydrase at 3 mg/mL (29 kDa) plus 250 μL of alcohol dehydrogenase at 5 mg/mL (150 kDa); 500 μL of bovine serum albumin (66 kDa); 500 μL of blue dextran (2000 kDa). The elution volume of blue dextran was used for the void volume of the column (*V*_0_). The *V*_e_ of the standards was determined, and the calibration curve was plotted as the log of the molecular mass versus *V*_e_/*V*_0_. The linear equation was then used to calculate the experimental molecular weight of each sample. The interaction was judged both by the calculated experimental size of each of the peaks as well as by a shift in the *V*_e_. The presence of both LIAS and NFU1 was confirmed by SDS–PAGE.

#### Isothermal Titration Calorimetry

ITC was employed to confirm the interaction between LIAS and NFU1 observed using the gel filtration method above and to determine the binding dissociation constant (*K*_D_). The *K*_D_ by ITC was determined by 2 μL injections of LIAS (150 μM in the syringe) into a solution of NFU1 (19 μM in the cell) at 27 °C using a MicroCal VP-ITC calorimeter (Malvern Pananalytical Ltd.) housed in an anaerobic chamber (<1 ppm of O_2_). Prior to the binding experiment, protein samples were thoroughly exchanged into ITC buffer (0.1 M HEPES pH 7.5, 0.5 M KCl, 5% glycerol, and 2 mM TCEP) by gel filtration chromatography using a PD-10 column. Binding analysis was accompanied by a control experiment in which LIAS ligand (150 μM) was titrated into the sample cell containing only the ITC buffer. Before the data were fit, the control raw data were subtracted from the corresponding raw titration data to account for the heat associated with ligand dilution. The corrected data were processed with the Origin 7 software package (Malvern Pananalytical Ltd.).

### Liquid Chromatography–Mass Spectrometry Activity Assays

Activity measurements were conducted in a Coy anaerobic chamber. Each reaction mixture contained the following at their final concentrations (unless noted otherwise elsewhere): 50 mM HEPES, pH 7.5, 250 mM KCl, 10 μM LIAS, 10 μM SAH nucleosidase, 0.75 mM SAM, and 350 μM octanoyllysyl-containing peptide substrate [Glu-(N^6^-octanoyl)Lys-Ala-Tyr]. In reactions designed to assess the effect of BOLA3, ISCA1, ISCA2, ISCU, GLRX5, or NFUI, 200 μM (final concentration) of each protein was added individually or in combination as needed. For reactions in which GLRX5 was added, reduced glutathione was included to a final concentration of 1 mM. For reactions designed to test direct cluster transfer from NFU1 to LIAS during turnover, sodium citrate was included to final concentration of 5 mM. All reactions were initiated by the addition of 1 mM (final concentration) sodium dithionite, and 25 μL aliquots were removed at various times and added to an equal volume of quench solution that contained 300 mM H_2_SO_4_, 8 mM TCEP, and 10 μM AtsA peptide external standard. The samples were centrifuged at 14 000*g* for 30 min to remove any precipitated proteins. The time-dependent formation of 6-thiooctanoyl intermediate and lipoyl peptide products was determined by UPLC–MS/MS using multiple reaction monitoring (MRM) (Table S3). The quenched assay mixture was separated on an Agilent Technologies Zorbax Extend-C18 column Rapid Resolution HT (4.6 mm × 50 mm, 1.8 μm particle size) equilibrated in 98% solvent A (0.1% formic acid, pH 2.6) and 2% solvent B (100% acetonitrile). A solvent gradient of 2–65% B was applied from 0.5 to 2.5 min, the solvent composition was maintained at 65% B for 0.5 min before being returned to 2% B in 1 min, and then was retained at 2% B for an additional 1 min to re-equilibrate the column. A flow rate of 0.3 mL/min was maintained throughout the method (Table S4). The detection of the products was performed using electrospray ionization in positive mode (ESI^+^) with the following parameters: nitrogen gas temperature of 300 °C and flow rate of 5.0 L/min, nebulizer pressure of 15 psi, and capillary voltage of 4000 V. The products were quantified based on standard curves of product standards run under same conditions.

## Supporting Information Available

DNA and protein sequences, Mössbauer and EPR spectra, activity plots, and LC–MS conditions (PDF)

## References

[ref1] Patterson E. L.; et al. Crystallization of a Derivative of Protogen-B. J. Am. Chem. Soc. 1951, 73 (12), 5919–5920. 10.1021/ja01156a566.

[ref2] Reed L. J.; et al. Crystalline α-Lipoic Acid: A Catalytic Agent Associated with Pyruvate Dehydrogenase. Science 1951, 114 (2952), 93 10.1126/science.114.2952.93. 14854913

[ref3] Reed L. Advances in Enzymology and Related Areas of Molecular Biology; John Wiley & Sons, Inc.: Hoboken, NJ, 2006; pp 319–347.

[ref4] Fujiwara K.; Okamura-Ikeda K.; Motokawa Y. Lipoylation of acyltransferase components of α-ketoacid dehydrogenase complexes. J. Biol. Chem. 1996, 271, 12932–12936. 10.1074/jbc.271.22.12932. 8662700

[ref5] Kang S. G.; Jeong H. K.; Lee E.; Natarajan S. Characterization of a lipoate-protein ligase A gene of rice (Oryza sativa L.). Gene 2007, 393 (1-2), 53–61. 10.1016/j.gene.2007.01.011. 17376611

[ref6] Schonauer M. S.; Kastaniotis A. J.; Kursu V. A.; Hiltunen J. K.; Dieckmann C. L. Lipoic acid synthesis and attachment in yeast mitochondria. J. Biol. Chem. 2009, 284, 23234–23242. 10.1074/jbc.M109.015594. 19570983 PMC2749097

[ref7] Spalding M. D.; Prigge S. T. Lipoic acid metabolism in microbial pathogens. Microbiology and molecular biology reviews: MMBR 2010, 74 (2), 200–228. 10.1128/MMBR.00008-10. 20508247 PMC2884412

[ref8] Cronan J. E. Biotin and Lipoic Acid: Synthesis, Attachment, and Regulation. EcoSal Plus 2014, 6 (1), 0001-2012 10.1128/ecosalplus.ESP-0001-2012. PMC4233344 26442940

[ref9] Ewald R.; et al. Lipoate-Protein Ligase and Octanoyltransferase Are Essential for Protein Lipoylation in Mitochondria of Arabidopsis. Plant Physiology 2014, 165 (3), 978–990. 10.1104/pp.114.238311. 24872381 PMC4081350

[ref10] Cronan J. E. Assembly of lipoic acid on its cognate enzymes: an extraordinary and essential biosynthetic pathway. Microbiol. Mol. Biol. Rev. 2016, 80, 429–450. 10.1128/MMBR.00073-15. 27074917 PMC4867368

[ref11] Solmonson A.; DeBerardinis R. J. Lipoic acid metabolism and mitochondrial redox regulation. J. Biol. Chem. 2018, 293, 7522–7530. 10.1074/jbc.TM117.000259. 29191830 PMC5961061

[ref12] Reed L. J. A trail of research from lipoic acid to -keto acid dehydrogenase complexes. J. Biol. Chem. 2001, 276, 38329–38336. 10.1074/jbc.R100026200. 11477096

[ref13] Mayr J. A.; Feichtinger R. G.; Tort F.; Ribes A.; Sperl W. Lipoic acid biosynthesis defects. J. Inherit. Metab. Dis. 2014, 37 (4), 553–563. 10.1007/s10545-014-9705-8. 24777537

[ref14] Habarou F.; Hamel Y.; Haack T. B.; Feichtinger R. G.; Lebigot E.; Marquardt; et al. Biallelic mutations in LIPT2 cause a mitochondrial lipoylation defect associated with severe neonatal encephalopathy. Am. J. Hum. Genet. 2017, 101, 283–290. 10.1016/j.ajhg.2017.07.001. 28757203 PMC5544388

[ref15] Cao X.; Zhu L.; Song X.; Hu Z.; Cronan J. E. Protein moonlighting elucidates the essential human pathway catalyzing lipoic acid assembly on its cognate enzymes. Proc. Natl. Acad. Sci. U. S. A. 2018, 115 (30), E7063–E7072. 10.1073/pnas.1805862115. 29987032 PMC6064980

[ref16] Parry R. J. Biosynthesis of lipoic acid. 1. Incorporation of specifically tritiated octanoic acid into lipoic acid. J. Am. Chem. Soc. 1977, 99 (19), 6464–6466. 10.1021/ja00461a061.

[ref17] White R. H. Stable isotope studies on the biosynthesis of lipoic acid in Escherichia coli. Biochemistry 1980, 19 (1), 15–19. 10.1021/bi00542a003. 6766313

[ref18] Jordan S. W.; Cronan J. E. Jr. A new metabolic link. J. Biol. Chem. 1997, 272, 17903–17906. 10.1074/jbc.272.29.17903. 9218413

[ref19] Jordan S. W.; Cronan J. E. [19] Biosynthesis of lipoic acid and posttranslational modification with lipoic acid in Escherichia coli. Methods Enzymol. 1997, 279, 176–183. 10.1016/S0076-6879(97)79021-9. 9211269

[ref20] Jordan S. W.; Cronan J. E. The Escherichia coli lipB Gene Encodes Lipoyl (Octanoyl)-Acyl Carrier Protein:Protein Transferase. J. Bacteriol. 2003, 185 (5), 1582–1589. 10.1128/JB.185.5.1582-1589.2003. 12591875 PMC148080

[ref21] Nesbitt N. M.; et al. Expression, purification, and physical characterization of Escherichia coli lipoyl(octanoyl)transferase. Protein Expr Purif 2005, 39 (2), 269–82. 10.1016/j.pep.2004.10.021. 15642479

[ref22] Parry R. J.; Trainor D. A. Biosynthesis of lipoic acid. 2. Stereochemistry of sulfur introduction at C-6 of octanoic acid. J. Am. Chem. Soc. 1978, 100 (16), 5243–5244. 10.1021/ja00484a073.

[ref23] Reed K. E.; Cronan J. E. Lipoic acid metabolism in Escherichia coli: sequencing and functional characterization of the lipA and lipB genes. J. Bacteriol. 1993, 175 (5), 1325–1336. 10.1128/jb.175.5.1325-1336.1993. 8444795 PMC193218

[ref24] Miller J. R.; et al. Escherichia coli LipA is a lipoyl synthase: in vitro biosynthesis of lipoylated pyruvate dehydrogenase complex from octanoyl-acyl carrier protein. Biochemistry 2000, 39, 15166–15178. 10.1021/bi002060n. 11106496

[ref25] Cicchillo R. M.; et al. Lipoyl synthase requires two equivalents of S-adenosyl-L-methionine to synthesize one equivalent of lipoic acid. Biochemistry 2004, 43 (21), 6378–86. 10.1021/bi049528x. 15157071

[ref26] Oberg N.; et al. RadicalSAM.org: A Resource to Interpret Sequence-Function Space and Discover New Radical SAM Enzyme Chemistry. ACS Bio & Med. Chem. Au 2022, 2 (1), 22–35. 10.1021/acsbiomedchemau.1c00048. PMC9477430 36119373

[ref27] Broderick J. B.; et al. Radical S-Adenosylmethionine Enzymes. Chem. Rev. 2014, 114 (8), 4229–4317. 10.1021/cr4004709. 24476342 PMC4002137

[ref28] Landgraf B. J.; McCarthy E. L.; Booker S. J. Radical S-Adenosylmethionine Enzymes in Human Health and Disease. Annu. Rev. Biochem. 2016, 85 (1), 485–514. 10.1146/annurev-biochem-060713-035504. 27145839

[ref29] Holliday G. L.; et al. Atlas of the Radical SAM Superfamily: Divergent Evolution of Function Using a ″Plug and Play″ Domain. Methods Enzymol 2018, 606, 1–71. 10.1016/bs.mie.2018.06.004. 30097089 PMC6445391

[ref30] Booker S. J.; Grove T. L. Mechanistic and functional versatility of radical SAM enzymes. F1000 Biol. Rep. 2010, 2, 52 10.3410/B2-52. 21152342 PMC2996862

[ref31] Bandarian V. Journey on the Radical SAM Road as an Accidental Pilgrim. ACS Bio Med Chem Au 2022, 10.1021/acsbiomedchemau.1c00059. PMC9204691 35726327

[ref32] Frey P. A.; Booker S. J. Radical mechanisms of S-adenosylmethionine-dependent enzymes. Adv. Protein Chem. 2001, 58, 1–45. 10.1016/S0065-3233(01)58001-8. 11665486

[ref33] Frey P. A.; Hegeman A. D.; Ruzicka F. J. The Radical SAM Superfamily. Crit Rev. Biochem Mol. Biol. 2008, 43 (1), 63–88. 10.1080/10409230701829169. 18307109

[ref34] Cicchillo R. M.; et al. Escherichia coli lipoyl synthase binds two distinct [4Fe–4S] clusters per polypeptide. Biochemistry 2004, 43, 11770–11781. 10.1021/bi0488505. 15362861

[ref35] Douglas P.; et al. Lipoyl synthase inserts sulfur atoms into an octanoyl substrate in a stepwise manner. Angew. Chem., Int. Ed. Engl. 2006, 45 (31), 5197–9. 10.1002/anie.200601910. 16835858

[ref36] Lanz N. D.; et al. Evidence for a catalytically and kinetically competent enzyme-substrate cross-linked intermediate in catalysis by lipoyl synthase. Biochemistry 2014, 53, 4557–4572. 10.1021/bi500432r. 24901788 PMC4216189

[ref37] Lanz N. D.; et al. Characterization of a radical intermediate in lipoyl cofactor biosynthesis. J. Am. Chem. Soc. 2015, 137, 13216–13219. 10.1021/jacs.5b04387. 26390103

[ref38] Lanz N. D.; Booker S. J. Auxiliary iron-sulfur cofactors in radical SAM enzymes. Biochim. Biophys. Acta 2015, 1853 (6), 1316–34. 10.1016/j.bbamcr.2015.01.002. 25597998

[ref39] Lanz N. D.; Booker S. J. The role of iron-sulfur clusters in the biosynthesis of the lipoyl cofactor. In Iron-Sulfur Clusters in Chemistry and Biology; Rouault T. A., Ed.; Walter de Gruyter GMbH: Berlin, Germany, 2014.

[ref40] Lanz N. D.; Booker S. J. Identification and function of auxiliary iron-sulfur clusters in radical SAM enzymes. Biochim. Biophys. Acta 2012, 1824 (11), 1196–212. 10.1016/j.bbapap.2012.07.009. 22846545

[ref41] Walsby C. J.; et al. An anchoring role for FeS clusters: chelation of the amino acid moiety of S-adenosylmethionine to the unique iron site of the [4Fe–4S] cluster of pyruvate formate–lyase activating enzyme. J. Am. Chem. Soc. 2002, 124, 11270–11271. 10.1021/ja027078v. 12236732

[ref42] Walsby C. J.; et al. Electron-nuclear double resonance spectroscopic evidence that S-adenosylmethionine binds in contact with the catalytically active [4Fe-4S]^+^ cluster of pyruvate formate-lyase activating enzyme. J. Am. Chem. Soc. 2002, 124, 3143–3151. 10.1021/ja012034s. 11902903

[ref43] Vey J. L.; Drennan C. L. Structural Insights into Radical Generation by the Radical SAM Superfamily. Chem. Rev. 2011, 111 (4), 2487–2506. 10.1021/cr9002616. 21370834 PMC5930932

[ref44] Harmer J. E.; et al. Structures of lipoyl synthase reveal a compact active site for controlling sequential sulfur insertion reactions. Biochem. J. 2014, 464, 123–133. 10.1042/BJ20140895. 25100160

[ref45] McLaughlin M. I.; et al. Crystallographic snapshots of sulfur insertion by lipoyl synthase. Proc. Natl. Acad. Sci. U. S. A. 2016, 113 (34), 9446–50. 10.1073/pnas.1602486113. 27506792 PMC5003258

[ref46] McCarthy E. L.; et al. The A-type domain in Escherichia coli NfuA is required for regenerating the auxiliary [4Fe-4S] cluster in Escherichia coli lipoyl synthase. J. Biol. Chem. 2019, 294 (5), 1609–1617. 10.1074/jbc.RA118.006171. 30538130 PMC6364782

[ref47] McCarthy E. L.; Booker S. J. Destruction and reformation of an iron-sulfur cluster during catalysis by lipoyl synthase. Science 2017, 358 (6361), 373–377. 10.1126/science.aan4574. 29051382 PMC5941298

[ref48] Maio N.; Rouault T. A. Iron-sulfur cluster biogenesis in mammalian cells: New insights into the molecular mechanisms of cluster delivery. Biochim. Biophys. Acta 2015, 1853 (6), 1493–512. 10.1016/j.bbamcr.2014.09.009. 25245479 PMC4366362

[ref49] Majewska J.; et al. Binding of the Chaperone Jac1 Protein and Cysteine Desulfurase Nfs1 to the Iron-Sulfur Cluster Scaffold Isu Protein Is Mutually Exclusive. J. Biol. Chem. 2013, 288 (40), 29134–29142. 10.1074/jbc.M113.503524. 23946486 PMC3790012

[ref50] Vickery L. E.; Cupp-Vickery J. R. Molecular chaperones HscA/Ssq1 and HscB/Jac1 and their roles in iron-sulfur protein maturation. Crit Rev. Biochem Mol. Biol. 2007, 42 (2), 95–111. 10.1080/10409230701322298. 17453917

[ref51] Fox N. G.; et al. The Human Iron-Sulfur Assembly Complex Catalyzes the Synthesis of [2Fe-2S] Clusters on ISCU2 That Can. Be Transferred to Acceptor Molecules. Biochemistry 2015, 54 (25), 3871–9. 10.1021/bi5014485. 26016389 PMC4675461

[ref52] Braymer J. J.; Lill R. Iron-sulfur cluster biogenesis and trafficking in mitochondria. J. Biol. Chem. 2017, 292 (31), 12754–12763. 10.1074/jbc.R117.787101. 28615445 PMC5546016

[ref53] Braymer J. J.; et al. Mechanistic concepts of iron-sulfur protein biogenesis in Biology. Biochim Biophys Acta Mol. Cell Res. 2021, 1868 (1), 118863 10.1016/j.bbamcr.2020.118863. 33007329

[ref54] Pérard J.; Ollagnier de Choudens S. Iron–sulfur clusters biogenesis by the SUF machinery: close to the molecular mechanism understanding. JBIC Journal of Biological Inorganic Chemistry 2018, 23 (4), 581–596. 10.1007/s00775-017-1527-3. 29280002 PMC6006206

[ref55] Rouault T. A.; Maio N. Biogenesis and functions of mammalian iron-sulfur proteins in the regulation of iron homeostasis and pivotal metabolic pathways. J. Biol. Chem. 2017, 292 (31), 12744–12753. 10.1074/jbc.R117.789537. 28615439 PMC5546015

[ref56] Rouault T. A. Biogenesis of iron-sulfur clusters in mammalian cells: new insights and relevance to human disease. Dis Model Mech 2012, 5 (2), 155–64. 10.1242/dmm.009019. 22382365 PMC3291637

[ref57] Banci L.; et al. [2Fe-2S] cluster transfer in iron–sulfur protein biogenesis. Proc. Natl. Acad. Sci. U. S. A. 2014, 111 (17), 6203 10.1073/pnas.1400102111. 24733926 PMC4035983

[ref58] Nasta V.; et al. A pathway for assembling [4Fe-4S](2+) clusters in mitochondrial iron-sulfur protein biogenesis. Febs j 2020, 287 (11), 2312–2327. 10.1111/febs.15140. 31724821

[ref59] Baker P. R.; Friederich M. W.; Swanson M. A.; Shaikh T.; Bhattacharya K.; Scharer G. H.; Aicher J.; Creadon-Swindell G.; Geiger E.; MacLean K. N.; et al. Variant non ketotic hyperglycinemia is caused by mutations in LIAS, BOLA3 and the novel gene GLRX5. Brain 2014, 137 (2), 366–379. 10.1093/brain/awt328. 24334290 PMC3914472

[ref60] Maio N.; Jain A.; Rouault T. A. Mammalian iron-sulfur cluster biogenesis: Recent insights into the roles of frataxin, acyl carrier protein and ATPase-mediated transfer to recipient proteins. Curr. Opin Chem. Biol. 2020, 55, 34–44. 10.1016/j.cbpa.2019.11.014. 31918395 PMC7237328

[ref61] Ahting U.; Mayr J. A.; Vanlander A. V.; Hardy S. A.; Santra S.; Makowski C.; Alston C. L.; Zimmermann F. A.; Abela L.; Plecko B.; et al. Clinical, biochemical, and genetic spectrum of seven patients with NFU1 deficiency. Front. Genet. 2015, 6, 123 10.3389/fgene.2015.00123. 25918518 PMC4394698

[ref62] Cameron J.; et al. Mutations in Iron-Sulfur Cluster Scaffold Genes NFU1 and BOLA3 Cause a Fatal Deficiency of Multiple Respiratory Chain and 2-Oxoacid Dehydrogenase Enzymes. American journal of human genetics 2011, 89, 486–95. 10.1016/j.ajhg.2011.08.011. 21944046 PMC3188835

[ref63] Navarro-Sastre A.; et al. A fatal mitochondrial disease is associated with defective NFU1 function in the maturation of a subset of mitochondrial Fe-S proteins. Am. J. Hum. Genet. 2011, 89 (5), 656–67. 10.1016/j.ajhg.2011.10.005. 22077971 PMC3213398

[ref64] Wachnowsky C.; et al. Understanding the Molecular Basis of Multiple Mitochondrial Dysfunctions Syndrome 1 (MMDS1)-Impact of a Disease-Causing Gly208Cys Substitution on Structure and Activity of NFU1 in the Fe/S Cluster Biosynthetic Pathway. J. Mol. Biol. 2017, 429 (6), 790–807. 10.1016/j.jmb.2017.01.021. 28161430 PMC5466808

[ref65] Melber A.; Na U.; Vashisht A.; Weiler B. D; Lill R.; Wohlschlegel J. A; Winge D. R Role of Nfu1 and Bol3 in iron-sulfur cluster transfer to mitochondrial clients. Elife 2016, 5, e15991 10.7554/eLife.15991. 27532773 PMC5014551

[ref66] Zhao S.; et al. Assembly of the covalent linkage between lipoic acid and its cognate enzymes. Chem. Biol. 2003, 10, 1293–1302. 10.1016/j.chembiol.2003.11.016. 14700636

[ref67] Billgren E. S.; Cicchillo R. M.; Nesbitt N. M.; Booker S. J. Lipoic acid biosynthesis and enzymology. In Comprehensive Natural Products II Chemistry and Biology; Mander L., Liu H.-W., Eds.; Elsevier: Oxford, U.K., 2010; pp 181–212.

[ref68] Cicchillo R. M.; Booker S. J. Mechanistic investigations of lipoic acid biosynthesis in Escherichia coli: both sulfur atoms in lipoic acid are contributed by the same lipoyl synthase polypeptide. J. Am. Chem. Soc. 2005, 127, 2860–2861. 10.1021/ja042428u. 15740115

[ref69] Cicchillo R. M.; et al. Lipoyl synthase requires two equivalents of S-adenosyl-L-methionine to synthesize one equivalent of lipoic acid. Biochemistry 2004, 43, 6378–6386. 10.1021/bi049528x. 15157071

[ref70] Douglas P.; et al. Lipoyl synthase inserts sulfur atoms into an octanoyl substrate in a stepwise manner. Angew. Chem. 2006, 118, 5321–5323. 10.1002/ange.200601910. 16835858

[ref71] McLaughlin M. I.; et al. Crystallographic snapshots of sulfur insertion by lipoyl synthase. Proc. Natl. Acad. Sci. U S A 2016, 113, 9446–9450. 10.1073/pnas.1602486113. 27506792 PMC5003258

[ref72] Lanz N. D.; et al. Characterization of Lipoyl Synthase from Mycobacterium tuberculosis. Biochemistry 2016, 55 (9), 1372–83. 10.1021/acs.biochem.5b01216. 26841001

[ref73] Camponeschi F.; et al. Paramagnetic (1)H NMR Spectroscopy to Investigate the Catalytic Mechanism of Radical S-Adenosylmethionine Enzymes. J. Mol. Biol. 2019, 431 (22), 4514–4522. 10.1016/j.jmb.2019.08.018. 31493409

[ref74] Hendricks A. L.; et al. Characterization and Reconstitution of Human Lipoyl Synthase (LIAS) Supports ISCA2 and ISCU as Primary Cluster Donors and an Ordered Mechanism of Cluster Assembly. Int. J. Mol. Sci. 2021, 22 (4), 1598 10.3390/ijms22041598. 33562493 PMC7915201

[ref75] Jain A.; et al. Assembly of the [4Fe-4S] cluster of NFU1 requires the coordinated donation of two [2Fe-2S] clusters from the scaffold proteins, ISCU2 and ISCA1. Hum. Mol. Genet. 2020, 29 (19), 3165–3182. 10.1093/hmg/ddaa172. 32776106 PMC7689295

[ref76] Johnson D. C.; Unciuleac M.-C.; Dean D. R. Controlled Expression and Functional Analysis of Iron-Sulfur Cluster Biosynthetic Components within Azotobacter vinelandii. J. Bacteriol. 2006, 188 (21), 7551–7561. 10.1128/JB.00596-06. 16936042 PMC1636278

[ref77] Pandelia M. E.; et al. Mössbauer spectroscopy of Fe/S proteins. Biochim. Biophys. Acta - Molecular Cell Research 2015, 1853 (6), 1395–1405. 10.1016/j.bbamcr.2014.12.005. 25498248

[ref78] Cai K.; et al. Structural/Functional Properties of Human NFU1, an Intermediate [4Fe-4S] Carrier in Human Mitochondrial Iron-Sulfur Cluster Biogenesis. Structure 2016, 24 (12), 2080–2091. 10.1016/j.str.2016.08.020. 27818104 PMC5166578

[ref79] Wachnowsky C.; et al. Regulation of human Nfu activity in Fe-S cluster delivery-characterization of the interaction between Nfu and the HSPA9/Hsc20 chaperone complex. Febs j 2018, 285 (2), 391–410. 10.1111/febs.14353. 29211945 PMC5777896

[ref80] Wesley N. A.; Wachnowsky C.; Fidai I.; Cowan J. A.; et al. Understanding the molecular basis for multiple mitochondrial dysfunctions syndrome 1 (MMDS1): impact of a disease-causing Gly189Arg substitution on NFU1. FEBS J. 2017, 284, 3838–3848. 10.1111/febs.14271. 28906594 PMC5696030

[ref81] Jumper J.; et al. Highly accurate protein structure prediction with AlphaFold. Nature 2021, 596 (7873), 583–589. 10.1038/s41586-021-03819-2. 34265844 PMC8371605

[ref82] Cai K.; Frederick R. O.; Markley J. L. ISCU interacts with NFU1, and ISCU[4Fe-4S] transfers its Fe-S cluster to NFU1 leading to the production of holo-NFU1. J. Struct Biol. 2020, 210 (2), 107491 10.1016/j.jsb.2020.107491. 32151725 PMC7261492

[ref84] Uzarska M. A; Nasta V.; Weiler B. D; Spantgar F.; Ciofi-Baffoni S.; Saviello M. R.; Gonnelli L.; Muhlenhoff U.; Banci L.; Lill R. Mitochondrial Bol1 and Bol3 function as assembly factors for specific iron-sulfur proteins. Elife 2016, 5, e16673 10.7554/eLife.16673. 27532772 PMC5014550

[ref85] Sheftel A. D.; et al. The human mitochondrial ISCA1, ISCA2, and IBA57 proteins are required for [4Fe-4S] protein maturation. Mol. Biol. Cell 2012, 23 (7), 1157–66. 10.1091/mbc.e11-09-0772. 22323289 PMC3315811

[ref86] Beinert H.; Holm R. H.; Münck E. Iron-sulfur clusters: nature’s modular, multipurpose structures. Science 1997, 277, 653–659. 10.1126/science.277.5326.653. 9235882

[ref87] Johnson M. K. Iron–Sulfur Proteins: New Roles for Old Clusters. Curr. Opin. Chem. Biol. 1998, 2, 173–181. 10.1016/S1367-5931(98)80058-6. 9667933

[ref88] Honarmand Ebrahimi K.; et al. Iron-sulfur clusters as inhibitors and catalysts of viral replication. Nat. Chem. 2022, 14 (3), 253–266. 10.1038/s41557-021-00882-0. 35165425

[ref89] Pritts J. D.; Michel S. L. J. Fe-S clusters masquerading as zinc finger proteins. J. Inorg. Biochem 2022, 230, 111756 10.1016/j.jinorgbio.2022.111756. 35247854

[ref90] Cameron J. M.; Janer A.; Levandovskiy V.; MacKay N.; Rouault T. A.; Tong W.-H.; Ogilvie I.; Shoubridge E. A.; Robinson B. H.; et al. Mutations in iron-sulfur cluster scaffold genes NFU1 and BOLA3 cause a fatal deficiency of multiple respiratory chain and 2-oxoacid dehydrogenase enzymes. Am. J. Hum. Genet. 2011, 89, 486–495. 10.1016/j.ajhg.2011.08.011. 21944046 PMC3188835

[ref91] Lossos A.; et al. Fe/S protein assembly gene IBA57 mutation causes hereditary spastic paraplegia. Neurology 2015, 84 (7), 659–67. 10.1212/WNL.0000000000001270. 25609768

[ref92] Debray F. G.; et al. Mutation of the iron-sulfur cluster assembly gene IBA57 causes fatal infantile leukodystrophy. J. Inherit Metab Dis 2015, 38 (6), 1147–53. 10.1007/s10545-015-9857-1. 25971455

[ref93] Iwig D. F.; Booker S. J. Insight into the polar reactivity of the onium chalcogen analogues of S-adenosyl-L-methionine. Biochemistry 2004, 43 (42), 13496–13509. 10.1021/bi048693+. 15491157

[ref94] Lanz N. D.; et al. RlmN and AtsB as models for the overproduction and characterization of radical SAM proteins. Methods Enzymol. 2012, 516, 125–152. 10.1016/B978-0-12-394291-3.00030-7. 23034227

[ref95] Bradford M. A rapid and sensitive method for the quantitation of microgram quantities of protein utilizing the principle of protein dye-binding. Anal. Biochem. 1976, 72, 248–254. 10.1016/0003-2697(76)90527-3. 942051

[ref96] Beinert H. Micro methods for the quantitative determination of iron and copper in biological material. Methods Enzymol. 1978, 54, 435–445. 10.1016/S0076-6879(78)54027-5. 732579

[ref97] Beinert H. Semi-micro methods for analysis of labile sulfide and of labile sulfide plus sulfane sulfur in unusually stable iron-sulfur proteins. Anal. Biochem. 1983, 131, 373–378. 10.1016/0003-2697(83)90186-0. 6614472

[ref98] Kennedy M. C.; et al. Evidence for the Formation of a Linear [3Fe-4S] Cluster in Partially Unfolded Aconitase. J. Biol. Chem. 1984, 259 (23), 14463–14471. 10.1016/S0021-9258(17)42622-6. 6094558

[ref99] Blaszczyk A. J.; et al. Spectroscopic and Electrochemical Characterization of the Iron-Sulfur and Cobalamin Cofactors of TsrM, an Unusual Radical S-Adenosylmethionine Methylase. J. Am. Chem. Soc. 2016, 138 (10), 3416–3426. 10.1021/jacs.5b12592. 26841310

