# Supplementary material for: In Vitro Demonstration of Human Lipoyl Synthase Catalytic Activity in the Presence of NFU1
Source: ACS Bio Med Chem Au. 2022 Jun 13;2(5):456–68. doi: 10.1021/acsbiomedchemau.2c00020 (PMC9585516; doi:10.1021/acsbiomedchemau.2c00020)
Supplement: Supplementary file 1 — bg2c00020_si_001.pdf [file bg2c00020_si_001.pdf]

Supporting Information for

In vitro Demonstration of Human Lipoyl Synthase (LIAS) Catalytic Activity in the Presence of NFU1

Douglas M. Warui[‡], Debangsu Sil[‡], Kyung-Hoon Lee[‡], Syam Sundar Neti[‡], Olga A. Esakova[‡], Hayley L. Knox, Carsten Krebs[,‡,§], and Squire J. Booker*[,‡,§,#]*

Departments of [‡]Chemistry and of [§]Biochemistry and Molecular Biology, and the [#]Howard Hughes Medical Institute, The Pennsylvania State University, University Park, Pennsylvania 16802, USA.

*To whom correspondence should be addressed: Squire J. Booker (sjb14@psu.edu); Carsten Krebs (ckrebs@psu.edu)

Table of Contents

1) DNA and protein sequences used in this study………….…………………..………………...… S3-S7

2) Mössbauer spectra of LIAS………………………………………..……………...…………....S8

3) Parameters used to simulate Mossbauer spectra of the $[3Fe\text{-}4S]^0$ Cluster ……...……….….……….S9

4) EPR spectra of NFU1………………………………………………………………………..S9

5) Activity plots of LIAS, LIAS with NFU1, BOLA3, ISCU, ISCA1&ISCA2, GLRX5………..…...S10

6) LC-MS gradient and MRM conditions for product analysis……………………..………………..S11

7) References……………………………………………………………………………..S11

DNA and amino acid sequences

The following are the codon-optimized DNA and the corresponding amino acid sequences of the various proteins (without their respective mitochondrial targeting sequences as indicated respectively) used in this study.

BOLA3 isoform 1 (a. a 27 – 107) optimized DNA sequence (UniProtKB Q53S33)

GCAACCCAGACCGAAGGTGAACTGCGTGTTACCCAGATTCTGAAAGAAAAATTTCCGCGTG

CAACCGCCATTAAAGTTACCGATATTAGCGGTGGTTGTGGTGCAATGTATGAAATCAAAAT

CGAAAGCGAAGAATTTAAAGAAAAGCGTACCGTTCAGCAGCATCAGATGGTTAATCAGGC

ACTGAAAGAAGAGATCAAAGAAATGCATGGTTTGCGCATTTTTACCAGCGTTCCGAAACGT

BOLA3 protein sequence

A T Q T E G E L R V T Q I L K E K F P R A T A I K V T D I S G G C G A M Y E I K I E S E E F K E

K R T V Q Q H Q M V N Q A L K E E I K E M H G L R I F T S V P K R

ISCA1 isoform 1 (a. a 13 - 129) optimized DNA sequence (UniProtKB Q9BUE6)

GTTAGCAAACGTAAACTGCAGCCGACACGTGCAGCACTGACCCTGACACCGAGCGCAGTT

AACAAAATCAAACAGCTGCTGAAAGATAAACCGGAACATGTTGGTGTTAAAGTTGGTGTTC

GTACCCGTGGTTGTAATGGTCTGAGCTATACCCTGGAATATACCAAAACCAAAGGCGATTC

AGATGAAGAGGTTATTCAGGATGGTGTGCGTGTGTTTATTGAAAAAAAAGCACAGCTGACC

CTGCTGGGCACCGAAATGGATTATGTTGAAGATAAACTGAGCAGCGAGTTCGTGTTTAATA

ACCCGAACATTAAAGGCACCTGTGGTTGTGGTGAAAGCTTTAACATTTAA

ISCA1 protein sequence

VSKRKLQPTRAALTLTPSAVNKIKQLLKDKPEHVGVKVGVRTRGCNGLSYTLEYTKT

KGDSDEEVIQDGVRVFIEKKAQLTLLGTEMDYVEDKLSSEFVFNNPNIKGTCGCGE

SFNI

ISCA2 isoform 1 (9 – 154) optimized DNA sequence (UniProtKB Q86U28)

CTG ACC GCA GCA ACC CAG CGT GCA GTT ACC CCG TGG CCT CGT GGT CGT CTG CTG ACC GCA AGT

CTG GGT CCG CAG GCA CGT CGT GAA GCA AGC AGC AGC AGT CCG GAA GCC GGT GAA GGT CAG ATT

CGT CTG ACC GAT AGC TGT GTT CAG CGT CTG CTG GAA ATT ACC GAA GGT AGC GAA TTT CTG CGT

CTG CAG GTT GAA GGT GGT GGT TGT AGC GGT TTT CAG TAT AAA TTC AGC CTG GAT ACC GTG ATT

AAT CCG GAT GAT CGT GTT TTT GAA CAA GGT GGT GCA CGT GTT GTT GTT GAT AGC GAT AGC CTG

GCA TTT GTT AAA GGT GCA CAG GTT GAT TTT AGC CAA GAA CTG ATT CGT AGC AGC TTT CAG GTT CTG

AAT AAT CCG CAG GCG CAG CAG GGT TGT AGC TGT GGT AGC AGT TTT AGC ATT AAA CTG

ISCA2 protein sequence

LTAATQRAVTPWPRGRLLTASLGPQARREASSSSPEAGEGQIRLTDSCVQRLLEITEGSEFLRLQ

VEGGGCSGFQYKFSLDTVINPDDRVFEQGGARVVVDSDSLAFVKGAQVDFSQELIRSSFQVLN

NPQAQQGCSCGSSFSIKL

ISCU isoform 1 (a. a 35 – 167) optimized DNA sequence (UniProtKB Q9H1K1)

TATCATAAAAAAGTGGTGGATCACTACGAGAATCCGCGTAATGTTGGTAGCCTGGATAAAA

CCAGCAAAAATGTTGGCACCGGTCTGGTTGGTGCACCGGCATGTGGTGATGTTATGAAACT

GCAGATTCAGGTGGATGAGAAAGGCAAAATTGTTGATGCACGCTTTAAAACCTTTGGTTGT

GGTAGCGCAATTGCAAGCAGCAGCCTGGCAACCGAATGGGTTAAAGGTAAAACCGTTGAA

GAAGCACTGACCATCAAAAATACCGATATTGCCAAAGAACTGTGTCTGCCTCCGGTTAAAC

TGCATTGTAGCATGCTGGCAGAAGATGCAATTAAAGCAGCACTGGCAGATTACAAACTGAA

ACAAGAACCGAAAAAAGGCGAAGCCGAGAAAAAA

ISCU protein sequence

YHKKVVDHYENPRNVGSLDKTSKNVGTGLVGAPACGDVMKLQIQVDEKGKIVDARFKTFG

CGSAIASSSLATEWVKGKTVEEALTIKNTDIAKELCLPPVKLHCSMLAEDAIKAALADYKLKQE

PKKGEAEKK

GLRX5 (a. a 32 – 157) optimized DNA sequence (UniProtKB Q86SX6)

GCCGGTAGTGGTGCAGGCGGTGGCGGTAGCGCAGAACAGCTGGATGCACTGGTTAAAAAA

GATAAAGTTGTGGTGTTCCTGAAAGGTACACCGGAACAGCCGCAGTGTGGTTTTAGCAATG

CAGTTGTTCAGATTCTGCGTCTGCATGGTGTGCGTGATTATGCAGCATATAATGTTCTGGAT

GATCCGGAACTGCGTCAGGGTATTAAAGATTATAGCAATTGGCCGACCATTCCGCAGGTTT

ATCTGAATGGTGAATTTGTTGGTGGTTGCGATATTCTGCTGCAGATGCATCAGAATGGTGAT

CTGGTTGAAGAACTGAAAAAACTGGGTATTCATAGCGCACTGCTGGATGAAAAAAAAGAT

CAGGATAGCAAATA

GLRX5 protein sequence

AGSGAGGGGSAEQLDALVKKDKVVVFLKGTPEQPQCGFSNAVVQILRLHGVRDYAAYNVLD

DPELRQGIKDYSNWPTIPQVYLNGEFVGGCDILLQMHQNGDLVEELKKLGIHSALLDEKKDQD

SK

LIAS isoform 1 (a. a 28 – 372) optimized DNA sequence (UniProtKB O43766)

CTGAGCAGCCTGCCGGATAAAAAGAAAGAACTGCTGCAGAATGGTCCGGATCTGCAGGAT

TTTGTTAGCGGTGATCTGGCGATCGTAGCACCTGGGATGAGTATAAAGGTAATCTGAAACG

TCAGAAAGGTGAACGTCTGCGTCTGCCTCCGTGGCTGAAAACCGAAATTCCGATGGGTAAA

AACTACAACAAACTGAAAAATACCCTGCGCAATCTGAATCTGCATACCGTTTGTGAAGAAG

CACGTTGTCCGAATATTGGTGAATGTTGGGGTGGTGGTGAATATGCAACCGCAACCGCCAC

CATTATGCTGATGGGTGATACCTGTACACGTGGTTGTCGTTTTTGTAGCGTTAAAACCGCAC

GTAATCCGCCTCCGCTGGATGCAAGCGAACCGTATAATACCGCAAAAGCAATTGCCGAATG

GGGCTTAGATTATGTTGTTCTGACCAGCGTTGATCGTGATGATATGCCGGATGGTGGTGCA

GAACATATTGCAAAAACCGTTAGCTATCTGAAAGAACGCAATCCGAAAATTCTGGTTGAAT

GTCTGACACCGGATTTTCGTGGTGATCTGAAAGCCATTGAAAAAGTTGCACTGAGCGGTCT

GGATGTTTATGCACATAATGTTGAAACCGTGCCGGAACTGCAGAGCAAAGTTCGTGATCCG

CGTGCAAATTTTGATCAGAGCCTGCGTGTTCTGAAACATGCAAAAAAAGTTCAGCCGGATG

TGATTAGCAAAACCAGCATTATGTTAGGTCTGGGCGAAAATGATGAACAGGTTTATGCAAC

CATGAAAGCACTGCGTGAAGCAGATGTGGATTGTCTGACCCTGGGCCAGTATATGCAGCCG

ACACGTCGTCATCTGAAAGTTGAAGAATATATCACCCCTGAGAAGTTCAAGTATTGGGAAA

AAGTGGGTAATGAACTGGGCTTTCATTATACCGCAAGCGGTCCGCTGGTTCGTAGCAGCTA

TAAAGCCGGTGAATTTTTTCTGAAAAACCTGGTGGCAAAACGCAAAACCAAAGATCTG

LIAS protein sequence

LSSLPDKKKELLQNGPDLQDFVSGDLADRSTWDEYKGNLKRQKGERLRLPPWLKTEIPMGKN

YNKLKNTLRNLNLHTVCEEARCPNIGECWGGGEYATATATIMLMGDTCTRGCRFCSVKTARN

PPPLDASEPYNTAKAIAEWGLDYVVLTSVDRDDMPDGGAEHIAKTVSYLKERNPKILVECLTPD

FRGDLKAIEKVALSGLDVYAHNVETVPELQSKVRDPRANFDQSLRVLKHAKKVQPDVISKTSI

MLGLGENDEQVYATMKALREADVDCLTLGQYMQPTRRHLKVEEYITPEKFKYWEKVGNELG

FHYTASGPLVRSSYKAGEFFLKNLVAKRKTKDL

NFU1 (a. a 59 – 254) optimized DNA sequence (UniProtKB Q9UMS0)

TTTATTCAGACCCAGGATACCCCGAATCCGAATAGCCTGAAATTCATTCCGGGTAAACCGG

TTCTGGAAACCCGTACCATGGATTTTCCGACACCGGCAGCAGCATTTCGTAGTCCGCTGGC

ACGTCAGCTGTTTCGTATTGAAGGTGTTAAAAGCGTTTTTTTCGGTCCGGATTTTATCACCG

TGACCAAAGAAAATGAAGAACTGGATTGGAATCTGCTGAAACCGGATATTTATGCCACCAT

CATGGATTTCTTTGCAAGCGGTCTGCCGCTGGTTACCGAAGAAACCCCGAGCGGTGAAGCA

GGTAGCGAAGAGGATGATGAAGTTGTTGCAATGATTAAAGAACTGCTGGATACCCGTATTC

GTCCGACCGTTCAAGAGGATGGTGGTGATGTTATCTATAAAGGTTTTGAAGATGGCATCGT

GCAGCTGAAACTGCAGGGTAGCTGTACCAGCTGTCCGAGCAGCATTATTACCCTGAAAAAT

GGCATTCAGAACATGCTGCAGTTTTATATCCCGGAAGTGGAAGGTGTTGAACAGGTTATGG

ATGATGAGTCCGATGAAAAAGAAGCAAATAGCCCG

NFU1 protein sequence

FIQTQDTPNPNSLKFIPGKPVLETRTMDFPTPAAAFRSPLARQLFRIEGVKSVFFGPDFITVTKENE

ELDWNLLKPDIYATIMDFFASGLPLVTEETPSGEAGSEEDDEVVAMIKELLDTRIRPTVQEDGGD

VIYKGFEDGIVQLKLQGSCTSCPSSIITLKNGIQNMLQFYIPEVEGVEQVMDDESDEKEANSP

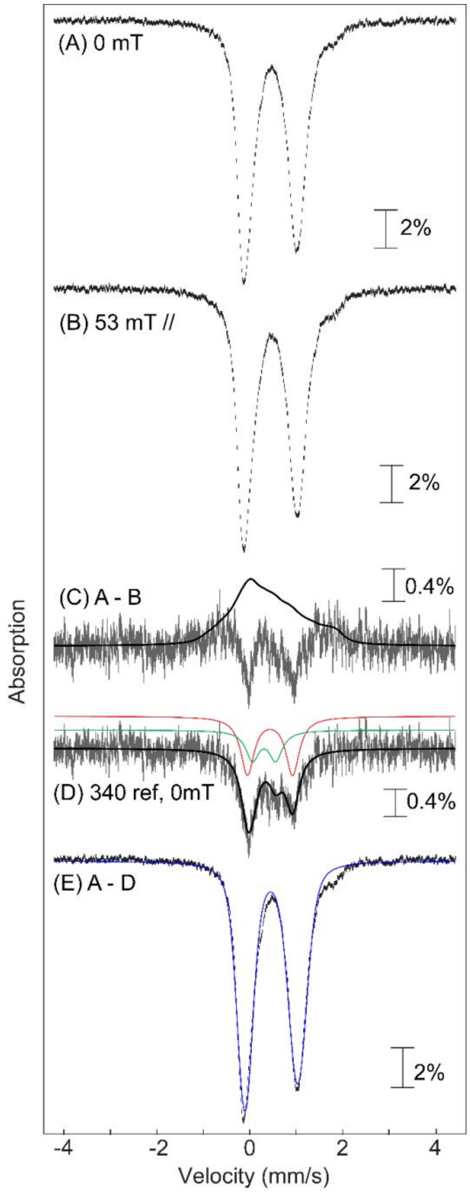

Figure S1. 4.2 K Mössbauer spectra of LIAS in (A) absence and (B) presence of a 53 mT external magnetic field applied parallel of the direction of propagation of γ beam. (C) [0 − 53 mT] difference spectrum (vertical bars), and simulation of $[3Fe\text{-}4S]^0$ cluster in 53 mT using the parameters listed in **Table S1** and scaled to ~ 10 % of total ^{57}Fe absorption (black line). (D) Zero-field reference spectrum of the $[3Fe\text{-}4S]^0$ cluster (vertical bars) generated by addition of the features of the $[3Fe\text{-}4S]^0$ cluster in a 53 mT field to the [0 − 53 mT] difference spectrum. Black line is the simulation of the $[3Fe\text{-}4S]^0$ cluster in zero field with parameters from **Table S2**. The red and green lines (in 2:1 intensity ratio) represents the

individual contributions from the three Fe sites. (E) Zero field spectrum of LIAS (vertical bars) after subtracting the features of the $[3Fe\text{-}4S]^0$ cluster (~ 10 %) from spectrum A. The blue line shows the features associated with the $[4Fe\text{-}4S]^{2+}$ cluster.

Table S1: Spin Hamiltonian Parameters for Simulation of Mössbauer Spectra of $[3Fe\text{-}4S]_0$ Clusters

ZFS parameters		δ (mm/s)	ΔE_Q (mm/s)	η	β(deg)[a]	$A/g_n\beta_n$ (T)
$D_{S=2} = -4.0$ cm^{-1}	Fe^{III}	0.31	-0.55	-2.0	16	(+10.0, +11.8, +11.8)
$(E/D)_{S=2} = 0.23$	$Fe_2^{2.5+}$	0.44	0.98	0.4	25	(-13.9, -13.9, -11.4)

[a]Euler angle β that rotates the electric field gradient tensor into the frame of the zero-field splitting tensor

Table S2. Different Sets of Mössbauer Parameters for Simulation of the Zero-Field Reference Spectrum of the $[3Fe\text{-}4S]^0$ Cluster

Site	δ (mm/s)	ΔE_Q (mm/s)
1 (trace D, Figure SX, green line)	0.31	0.50
2 (trace D, Figure SX, red line)	0.44	0.97

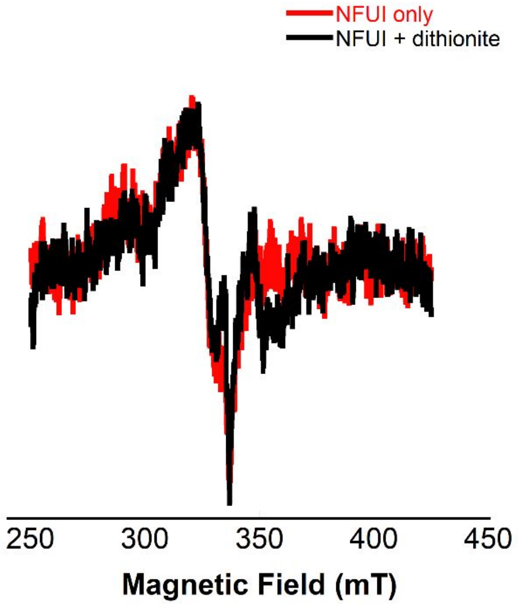

Figure S2. EPR spectra of 400 µM NFU1, unreduced (red), and reduced with 4 mM dithionite (black), showing no apparent differences or any distinctive features. The spectra were collected at 10 K, 10 mW microwave power, and 0.2 mT modulation amplitude.

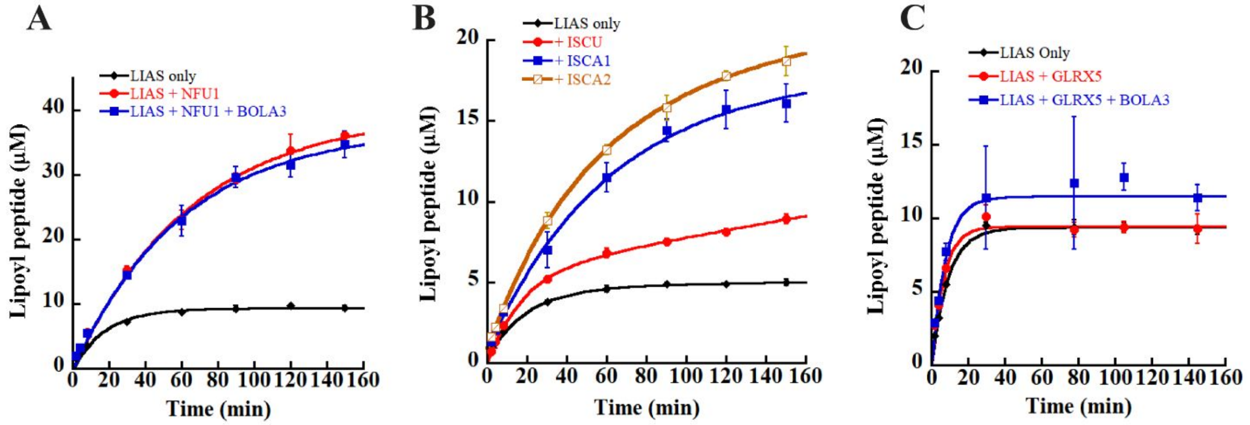

Figure S3. In vitro activity results of 10 µM LIAS; in the presence of 200 µM NFU1 and 200 µM NFU1 and 200 µM BOLA3 (**A**), in the presence of 200 µM ISCU, 200 µM ISCA1 or 200 µM ISCA2 (**B**), in the presence of 200 µM GLRX5 and 200 µM each of GLRX5 and BOLA3 (**C**). The inclusion of NFU1 (red trace) in the LIAS reaction allows for multiple turnovers of the lipoyl product while inclusion of BOLA3 (blue trace) in a reaction that also contained NFU1 showed no notable effect (**A**). When ISCU (red trace) was included in LIAS activity assays, a slight increase in lipoyl product to a full turnover was observed, inclusion of ISCA1 (blue trace) caused an increase in lipoyl product to ~ 0.5 additional turnover while the inclusion of ISCA2 (orange trace) led to an increase of lipoyl product to ~ 1 additional turnover (**B**). The inclusion of GLRX5 (red trace) alone or in combination with BOLA3 (blue trace) showed no observable effect on LIAS acitivity (**C**). Unless otherwise noted, all the activity assays included in their final concentrations; 350 µM octanoyl peptide substrate, 0.75 mM SAM and 10 µM SAH nucleosidase, and were initiated with 1 mM dithionite. The reactions were carried out at room temperature in a buffer that contained 50 mM HEPES pH 7.5 and 0.25 M KCl. For reactions in which GLRX5 was included, 1 mM

reduced glutathione was also added. The respective data shown in panels **A**, **B** and **C** are averages from assays done in triplicate and the associated standard deviation error bars are shown in the respective data traces. The 6-thiooctanoyl intermediate data were fit to an exponential equation that accounts for its formation and decay phases (Figure 7A and 7B) while the lipoyl peptide product data were fit to a biphasic double exponential rate of formation equation respectively assuming a A $\rightarrow$ B $\rightarrow$ C model, as has previously been reported for *Mycobacterium tuberculosis* LipA [72].

Table S3: MRM fragmentation products monitored by LC-MS.

Compound	Parent ion*	Product ion 1§	Product ion 2§
lipoyl peptide	700.2 (211)	274.1 (36)	84 (68)
$^{32}S/^{34}S$ lipoy peptide	702.2 (211)	276.1 (36)	84 (68)
$^{34}S/^{34}S$ lipoy peptide	704.2 (211)	278.1 (36)	84 (68)
monothiolated peptide	668.2 (211)	242.1 (32)	84 (68)
^{34}S monothiolated peptide	670.2 (211)	244.1 (32)	84 (68)
octanoyl peptide	636.3 (196)	210.1 (32)	84 (60)
AtsA peptide	474.4 (112)	229.1 (18)	153 (26)

*Respective fragmentor voltage in parenthesis

§ Respective collision energies in parenthesis

Table S4: LC-MS gradient conditions for the analysis of LIAS reaction products

Time (min)	0.1% Formic acid in water	Acetonitrile	Flow rate (mL/min)
0	98%	2%	0.3
0.5	98%	2%	0.3
2.5	35%	65%	0.3
3.0	35%	65%	0.3
4	98%	2%	0.3
5	98%	2%	0.3

References

1. Steinthorsdottir, V.; Thorleifsson, G.; Reynisdottir, I.; Benediktsson, R.; Jonsdottir, T.; Walters, G. B.; Styrkarsdottir, U.; Gretarsdottir, S.; Emilsson, V.; Ghosh, S.; Baker, A.; Snorradottir, S.; Bjarnason, H.; Ng, M. C.; Hansen, T.; Bagger, Y.; Wilensky, R. L.; Reilly, M. P.; Adeyemo, A.; Chen, Y.; Zhou, J.; Gudnason, V.; Chen, G.; Huang, H.; Lashley, K.; Doumatey, A.; So, W. Y.; Ma, R. C.; Andersen, G.; Borch-Johnsen, K.; Jorgensen, T.; van Vliet-Ostaptchouk, J. V.; Hofker, M. H.; Wijmenga, C.; Christiansen, C.; Rader, D. J.; Rotimi, C.; Gurney, M.; Chan, J. C.; Pedersen, O.; Sigurdsson, G.; Gulcher, J. R.; Thorsteinsdottir, U.; Kong, A.; Stefansson, K., A variant in CDKAL1 influences insulin response and risk of type 2 diabetes. *Nature genetics* **2007,** *39* (6), 770-5.
